# Uptake of Nanoparticles as a Model System for Viruses under the Presence of Chloroquine

**DOI:** 10.1021/acsnanoscienceau.5c00183

**Published:** 2026-01-23

**Authors:** Gan Chen, Dingcheng Zhu, Yaofeng Zhao, Timothy K. Soh, Ruixia Wang, Neus Feliu, Wolfgang J. Parak

**Affiliations:** † Center for Hybrid Nanostructures (CHyN), Universität Hamburg, 22607 Hamburg, Germany; ‡ Academy of Military Medical Sciences, Beijing 100850, China; § Centre for Structural Systems Biology, 22607 Hamburg, Germany; ∥ Hannover Medical School, Institute of Virology, 30625 Hannover, Germany; ⊥ Cluster of Excellence RESIST (EXC 2155), Hannover Medical School, 30625 Hannover, Germany; # Leibniz Institute of Virology (LIV), 20251 Hamburg, Germany; ∇ Deutsches Elektronen-Synchrotron DESY, 22607 Hamburg, Germany

**Keywords:** nanoparticle uptake, endocytosis, virus, chloroquine, endosomal escape

## Abstract

In the context of fighting SARS-CoV-2 infection, chloroquine and its derivatives have been suggested as treatments. It has been argued that the therapeutic effect is based on the modulation of viral uptake and fate. Similarities to the endocytosis of nanoparticles (NPs) are used here to recapitulate the effect of chloroquine on this process. The possibilities and limits of this approach are discussed. In particular, it is demonstrated that the uptake of NPs can be diminished in the presence of toxic chloroquine concentrations. Chloroquine further reduced acidification of endosomes/lysosomes, which may interfere with the optical readout of fluorescence-labeled NPs. Furthermore, depending on the system used, chloroquine can either increase or decrease transfection upon particle-based DNA delivery.

## Graphical abstract

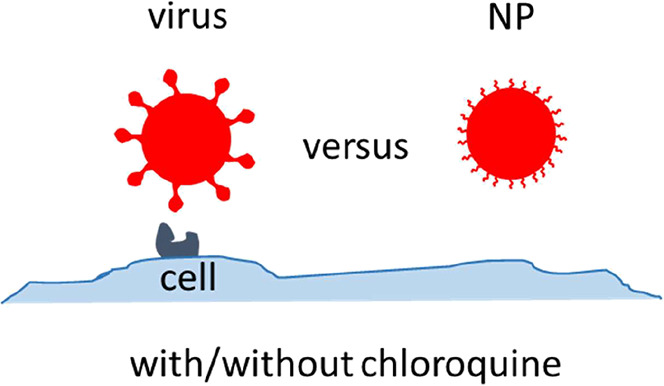

## Introduction

During the COVID-19 pandemic, there was a group of reports in which chloroquine and, in particular, its more water-soluble derivative hydroxychloroquine, were suggested as a treatment for COVID-19, i.e., the disease caused by SARS-CoV-2 infection.^1,2^ In such studies, it is frequently claimed that chloroquine modulates endocytosis and transfection. There are, in fact, many reports in which endocytosis is shown as a cellular entry route for the SARS-CoV-2 virus,^3–7^ apart from other possible entry routes, such as fusion with the plasma membrane. Endocytosis leads to entrapment of virions in endosomes/lysosomes, and thus, for transfection, endosomal escape/release is needed, which is, in general, true for gene delivery.^8^ In addition, there is a debate about the role of endosomal acidification in SARS-CoV-1/2 virus infection,^9,10^ and in this context, it is of importance that chloroquine may interfere with endosomal acidification.^11^ If one looks at this with the eyes of a colloid scientist, there are many similarities to the uptake of colloidal nanoparticles (NPs). Arguably, viruses and NPs have several physicochemical features in common.^12,13^ The relevance of NP uptake in the context of nanomedicine to SARS-CoV-2 virus infection was also highlighted in a recent comment.^2^ Thus, NPs could be used as a model system for investigating the effect of chloroquine on SARS-CoV-2 entry into cells.

To what extent NPs may mimic the cellular internalization process of SARS-CoV-2 depends on the specific internalization mechanisms of both. In the following, some key similarities and differences between the two are outlined. Concerning similarities, there is most importantly (i) the particulate nature. SARS-CoV-2 is a spherical virus with a diameter of 60–140 nm, exhibiting particle-like geometry.^14^ NPs typically cover the whole range from a few nanometers to a few hundred nanometers in size. (ii) The particulate nature facilitates endocytic uptake by cells. SARS-CoV-2 enters cells through receptor-mediated endocytosis after its S-protein binds to ACE2, resulting in entrapment by endosomes.^3–7^ NPs are also in general internalized via various endocytic pathways and thus are not free inside the cytosol but encapsulated in endosomes.^15,16^ (iii) There may be a pH-dependent endosomal escape. While not efficient, for both endocytosed SARS-CoV-2 and NPs, endosomal acidification may accelerate endosomal escape. There are, however, also remarkable differences. (i) Concerning the internalization mechanism, SARS-CoV-2 enters host cells via binding to the host receptor angiotensin-converting enzyme 2 (ACE2) and then undergoes spike (S) glycoprotein-mediated membrane fusion or endocytic uptake.^17,18^ Specifically, SARS-CoV-2 is a β-coronavirus, that incorporates its spike glycoprotein (S), envelope protein­(E), and membrane protein­(M) into its envelope. Its infection begins with the priming of S with cleavage by predominantly the host protease TMPRSS2, which is followed by binding of S to its receptor ACE2 to trigger membrane fusion and release of the viral genome into the cytoplasm. In the absence of TMPRSS2, the virus is internalized via endocytosis and encapsulated within endosomes,^19^ primarily involving clathrin-mediated endocytosis (CME).^6,20,21^ In contrast, NPs primarily enter cells through endocytosis, and the internalization mechanism varies depending on their size, surface modifications, and cell type, potentially involving macropinocytosis, phagocytosis, CME, or Caveolae-mediated endocytosis (CvME).^22^ (ii) There is also a different degree concerning the involvement of specific receptors. As reported, SARS-CoV-2 relies on its specific S protein binding to ACE2 and the activation of TMPRSS2 for infection.^23^ In contrast, NPs are endocytosed to a large degree nonspecifically, i.e., without the involvement of specific ligand–receptor interaction. Targeting of membrane-bound receptors may enhance NP uptake,^24^ but there is a “background” of nonspecific uptake. Nonspecific NP uptake is modulated to a certain degree by the physicochemical properties of the NPs, in particular, surface charge and size.^22,25,26^ (iii) Further differences can be found in the endosomal processing. SARS-CoV-2 can utilize host lysosomal proteases (such as Cathepsin L and B), which are dependent on endosomal acidification, to activate the S protein^27–29^ to mediate endosomal escape by fusion of the viral envelope with the endosomal membrane and release of RNA. In contrast, the endosomal escape of NPs relies on a variety of different mechanisms, such as pH-responsive chemical release, receptor-mediated escape, or physicochemical properties that disrupt the endosomal membrane.^30–32^ Unlike SARS-CoV-2, NPs do not depend on specific host proteases for endosomal escape. Proteases, however, may degrade NPs,^33,34^ leading to enhanced exocytosis.^26,35^ In general, endosomal escape of NPs is inefficient, which is an intrinsic problem for the development of nanomedicines.^36^

Thus, bare or encapsulated NPs, lacking surface ligands or receptors that emulate or mimic SARS-CoV-2 ligands, cannot describe the internalization process of SARS-CoV-2 in full detail. As mentioned, SARS-CoV-2 uses its S protein to interact with the cell surface as the first step in the infection process. After engagement with the plasma membrane, SARS-CoV-2 undergoes rapid, clathrin-mediated endocytosis, when the TMPRSS2 is absent.^6^ This process is not included in the endocytosis of the generic NPs. However, NPs may mimic aspects of SARS-CoV-2 internalization that depend on the particulate nature. For the interpretation of the following experimental investigation, it is highly important to keep these limitations in mind. In this way, in the following, the effect of chloroquine on the generic uptake of NPs and viruses, based on their particulate nature, neglecting ligand–receptor effects, will be investigated.

In order to discuss the effect of chloroquine on NP uptake and transfection, we show here a basic set of simple protocols, which allow for quantitative analysis. Such protocols are established in the NP community and will be put into the context of their relevance for viral infection. As there are many general statements in the literature, such as “chloroquine inhibits endocytosis of NPs”,^2^ we chose, on purpose, a generic standard system without specific features, such as ligand–receptor interactions, in the form of polymer-encapsulated Au NPs labeled with a fluorophore and HeLa cells. Note that while Hu et al. discuss clathrin-mediated endocytosis,^2^ nonspecific endocytosis also leads to the uptake of NPs and thus forms the “background” of receptor-mediated or clathrin-mediated endocytosis. If one waits long enough, most NPs in general will be endocytosed.^37^ NPs without specific surface ligands, thus, are the most general system. Having such a generic system allows us to quickly verify/falsify general claims, which do not refer to a particular surface coating, size, etc.

## Nanoparticle Uptake under the Influence of Chloroquine

Studying NP uptake by cells is not as straightforward as one may think, and everything depends on sufficient controls.^37^ We chose a very simple setup. Cells were always exposed to the same concentration *c*_NP_ ≈ 63 nM of fluorescence-labeled Au NPs with a core diameter of approximately 4 nm (the concentration was determined by measuring the elemental concentration of gold, and estimating the number of Au atoms *per* NP from the NP size as derived from transmission electron microscopy (TEM) pictures; for their characterization cf. Figure S1).^38^ Cells were treated with different concentrations *c*_CQ_ of chloroquine, and NP uptake was quantified by measuring the mean fluorescence *I*_NP_ of treated cells by flow cytometry with a filter set tailored to the emission of the NPs (Figure 1a_1_; the raw data are shown in the Supporting Information (SI), Figures S9–S11). The fluorescence *I*_NP_ at *c*_CQ_ = 0 comprises the fluorescence of NPs associated with cells and the autofluorescence of cells (which was, in this case, not removed by additional gating). Note that with this protocol, fluorescence of the NPs may involve some NPs that are only adsorbed to the outer cell membrane and have not been endocytosed yet.^39^ However, previous studies with similar NPs and cells, based on several methods, e.g., TEM analysis, colocalization, etc., have demonstrated that the majority of the NPs are endocytosed and thus located inside cells in endosomal/lysosomal vesicles.^40^ This is briefly demonstrated here by recording fluorescence microscopy z-stack images of cells incubated with NPs and chloroquine (Figure S7). There are some agglomerated NPs found outside cells (i.e., not in the focal plane of the cell bodies). The intracellular NPs show the typical grainy pattern of endocytosed NPs (Figures S7 and S8).

**Figure 1:**
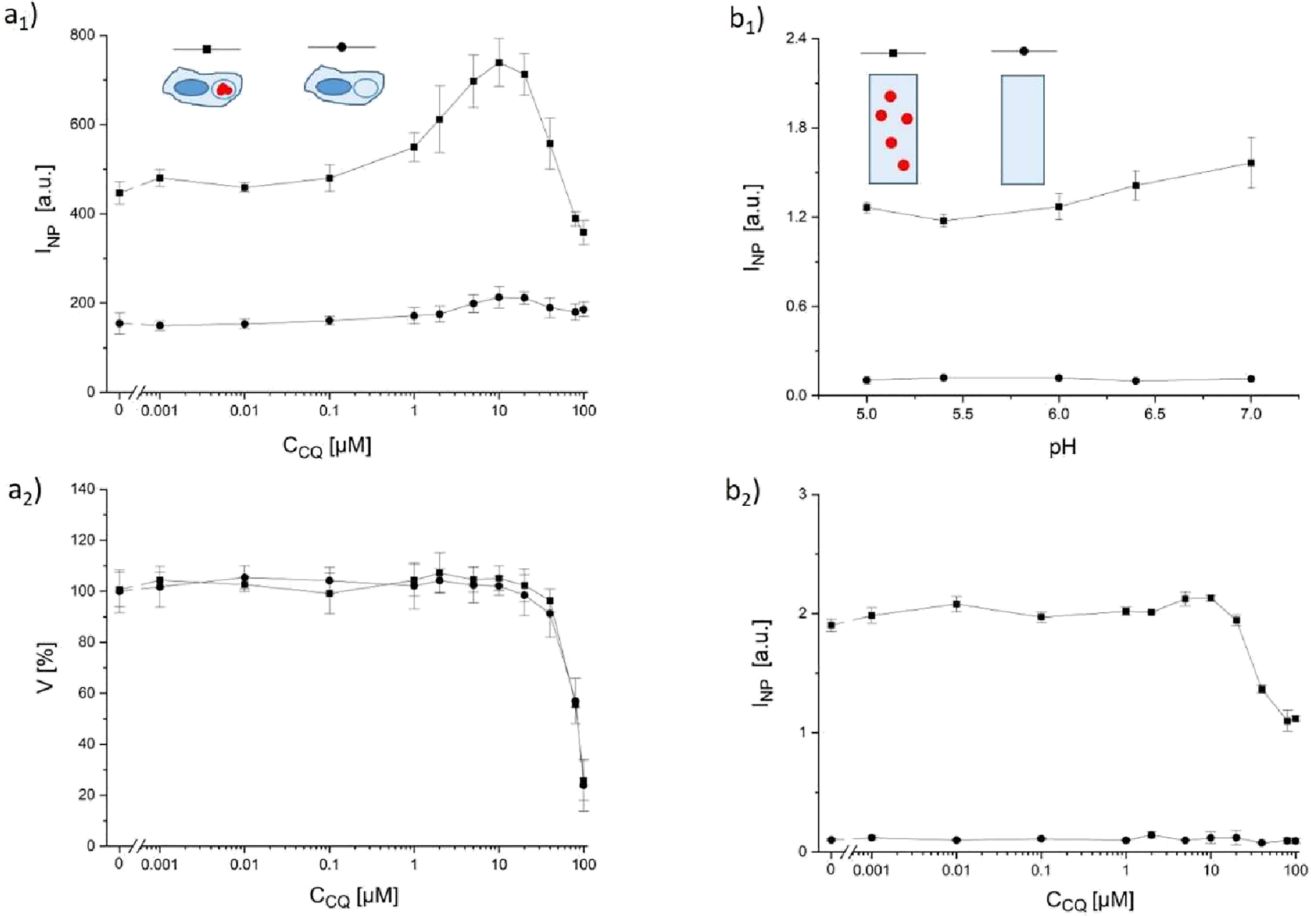
(a) Cells treated with different concentrations of chloroquine *c*_CQ_ were incubated with TAMRA-modified Au NPs. Cells without added Au NPs were used as a control. In (a_1_), the averaged fluorescence intensity *I*_NP_ per cell, as recorded with a flow cytometer with TAMRA fluorescence settings, is plotted versus the chloroquine concentration. In (a_2_), the corresponding cell viability V is given. (b) Cuvette measurements in which the fluorescence *I*_NP_ of the Au NPs was probed with a fluorimeter for the influence of (b_1_) pH and (b_2_) chloroquine. As a control, the fluorescence of the same solution but without Au NPs was recorded.

To measure the autofluorescence of chloroquine bound to cells, we also measured cells treated with chloroquine but not with NPs. The data in Figure 1a_1_ show that autofluorescence of cells is negligible. There is also some chloroquine fluorescence at concentrations *c*_CQ_ > 3 μM, which, however, is well below the fluorescence of the internalized Au NPs. Data show that with rising chloroquine concentrations, there is enhanced cellular fluorescence, which relates to a higher amount of internalized NPs. This is in contrast to the general statement that “chloroquine decreases the accumulation of synthetic NPs”.^2^ However, even higher concentrations of chloroquine, i.e., *c*_CQ_ > 10 μM, decrease fluorescence. Whereas this could suggest that there are few NPs in cells, in order to understand this effect, we also have to think of other explanations. It is known that the fluorescence of the Au NPs (i.e., the tetramethylrhodamine (TAMRA) embedded in the NP shell) depends on pH (Figure 1b_1_ and for the raw data Figure S3, i.e., at lower pH as in the case of NPs inside endosomes/lysosomes, the fluorescence goes down), as it is also quenched by high concentrations of chloroquine (Figure 1b_2_ and for the raw data Figures S4 and S5, for the fluorescence of chloroquine without NPs cf. Figure S6). Fluorescence quenching upon high concentrations of chloroquine could explain the decay of fluorescence at very high chloroquine concentrations (*c*_CQ_ > 10 μM) but not the raise in fluorescence for *c*_CQ_ > 1 μM (Figure 1a_1_). This could be due to the toxic side effects of chloroquine. Thus, we measured cell viability V for all exposure conditions, i.e., with/without NPs and at different chloroquine concentrations in the standard resazurin assay.^40^ Data in Figure 1a_2_ show that the presence of NPs did not affect cell viability (*c*_CQ_ = 0; *c*_NP_ = 0 versus *c*_NP_ = 63 nM). On the other hand, there was a massive reduction in cell viability due to the presence of chloroquine, starting at concentrations *c*_CQ_ > 10 μM. Note that cell viability is a very crude measure of toxicity, and in fact, impairment of cells may start at much lower doses.^40^ Comparing the uptake data with the viability data strongly suggests that reduced NP uptake at *c*_CQ_ > 10 μM is caused by the onset of toxic effects of chloroquine (Figure 1a_2_), and there is also chloroquine-induced fluorescence quenching (Figure 1b_2_). Given these data, a general statement that chloroquine, in general, reduces NP uptake is questionable. The data of Figure 1 are an example of increased NP uptake upon the presence of chloroquine (under nontoxic conditions). This would be an unwanted effect during the treatment of a viral infection.

## Endo/Lysosomal pH under the Influence of Chloroquine

After endocytic uptake, NPs reside in vesicular compartments, i.e., different stages of endosomes, lysosomes, etc*.* ^41–44^ Thus, one effect to be considered is that the presence of chloroquine reduces the acidification of endosomes/lysosomes.^45–47^ Increase of endo/lysosomal pH due to chloroquine can be conveniently measured by endocytosed pH-sensitive fluorophores.^48,49^ This is demonstrated in Figure 2. Cells were exposed to the same concentration of the encapsulated pH-sensitive fluorophore SNARF (for their characterization cf. Figure S2). The ratiometric fluorescence emission of SNARF can be related by a calibration curve (which needs to be done in perforated cells in order to take into account scattering effects) to the endosomal/lysosomal pH^37,49^ (Figure 2a; for the gating strategy and the raw data cf. Figures S12 and S13, respectively). As the encapsulated SNARF resides in lysosomes,^37,50,51^ its ratiometric fluorescence relates to the lysosomal pH,^49,50^ which can be traced back to the pH of individual intracellular vesicles.^50^ Autofluorescence of the cells was removed by appropriate gating. In Figure 2b_1_ (for the gating strategy and the raw data cf. Figures S14 and S15, respectively), it is shown how the acidic pH of lysosomes on a whole cell level (i.e., without lateral resolution within one cell) becomes less acidic with rising concentrations of chloroquine. This effect lasts until the onset of toxic effects of chloroquine (at *c*_CQ_ ≈ 10 μM), as shown in the viability assay (Figure 2b_2_). We can thus recapitulate the known fact that chloroquine neutralizes the acidic pH of lysosomes until, at high chloroquine concentrations, there is a reduction in cell viability.

**Figure 2:**
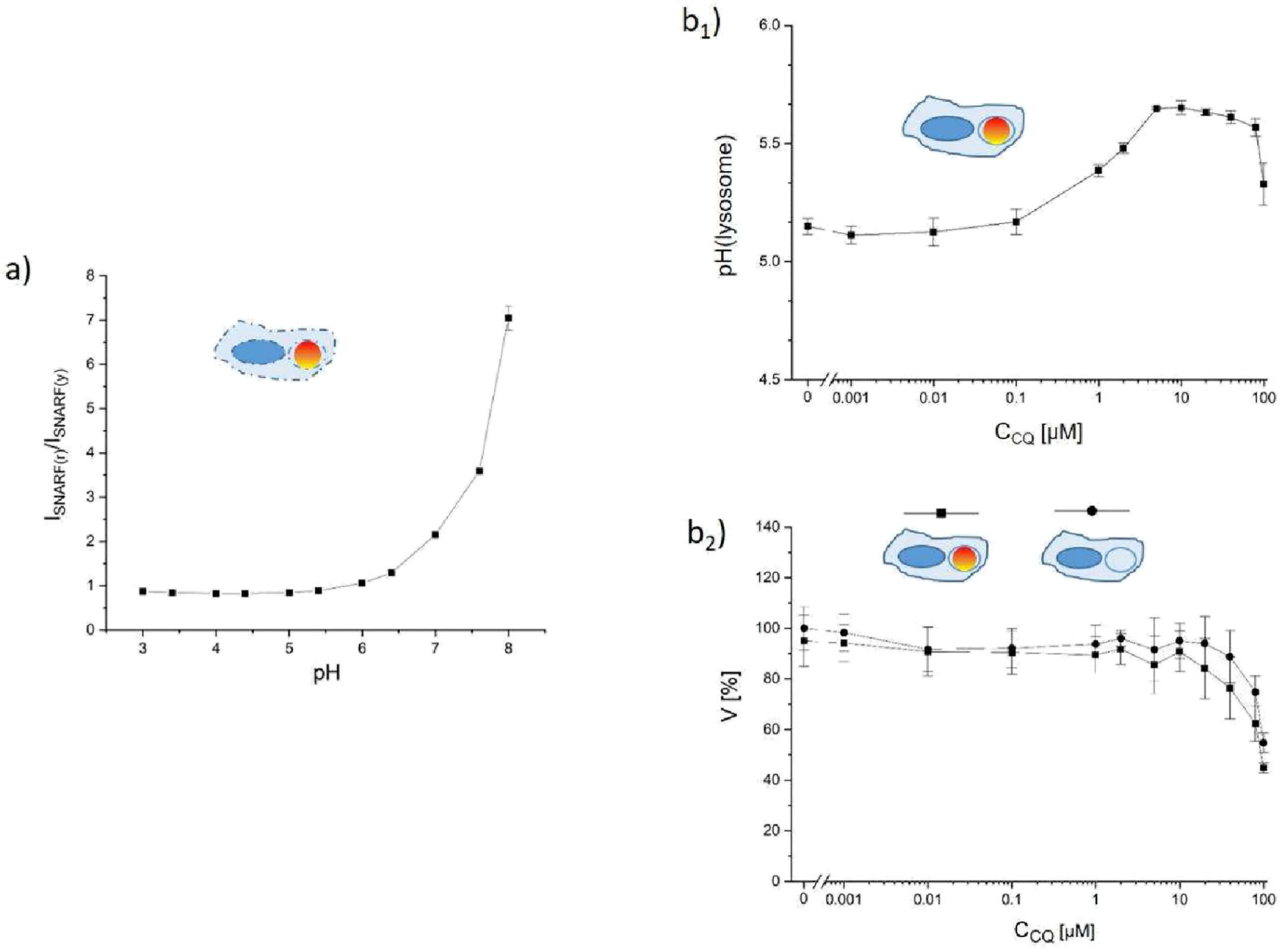
Lysosomal acidification and cell viability. (a) Calibration curve, in which the ratiometric fluorescence readout (red versus yellow fluorescence intensity; details of the filter settings are provided in the Supporting Information) of the pH-responsive fluorophore SNARF is plotted versus the pH, according to previous protocols.^50^ (b) Change in endosomal/lysosomal pH and in cell viability V upon different exposure conditions of chloroquine *c*_CQ_.

How does this affect transfection? There are contradicting reports in the literature, in which for some NP systems, transfection increased under the presence of chloroquine, but for others it decreased.^52,53^ We thus probed DNA transfection with three NP-based formulations to investigate whether the presence of chloroquine increased or decreased transfection. For this, three different delivery systems were used. Plasmids encoding eGFP (peGFP) were (i) complexed with polyethylene imine (PEI) to form polyplexes,^54^ (ii) embedded in biodegradable capsules,^55^ and (iii) complexed with lipofectamine to form lipoplexes.^56,57^ Polyplexes and capsules have been demonstrated to be endocytosed.^51,54^ As before, the concentration *c*_NP_ of the NP-based formulation, and thus the concentration of the plasmid, was kept constant, while the concentration of chloroquine was varied. Transfection was measured in terms of eGFP fluorescence *I*_eGFP_ *per* cell, as detected with a flow cytometer with filter settings adjusted to the fluorescence properties of eGFP. In order to compensate for the autofluorescence of cells (which in this case was not removed by gating) and the chloroquine fluorescence, measurements were repeated with cells that had not been exposed to the NP-based formulations. Data in Figure 3 (for the corresponding raw data cf. Figures S16–S21) show that the recorded fluorescence is due to expressed eGFP, and not due to autofluorescence of cells/fluorescence of chloroquine. For the polyplexes (Figure 3a_1_) and lipoplexes (Figure 3c_1_), transfection decreased with rising chloroquine concentration, whereas for the capsules (Figure 3b_1_), transfection first increased before decreasing upon increasing chloroquine concentrations. In order to understand this effect, the reduction in cell viability due to the presence of chloroquine was measured as a control (Figure 3a_2_,b_2_,c_2_). First, the presence of the delivery vehicle did not significantly reduce cell viability (*c*_CQ_ = 0, *c*_NP_ = 0 versus *c*_NP_ > 0). As already reported in Figures 1a_2_ and 2b_2_, for *c*_CQ_ > 10 μM, cell viability suffers. In fact, this is the chloroquine concentration at which transfection with lipoplexes severely goes down. Transfection with polyplexes partly decreases at lower chloroquine concentrations. In the case of the capsules, there is an increasing transfection up to the point where chloroquine impairs cell viability, and from this point on, transfection decreases, similar to the results of previous studies.^58,59^ While it is out of the scope of this work to pin down the mechanism for this, we speculate that the reduction of acidity in the endo- and lysosomes might improve endosomal escape. At any rate, there is at least one delivery scenario under which transfection increases in the presence of chloroquine under nontoxic conditions. Again, this actually would be an unwanted effect for reducing viral infection.

**Figure 3:**
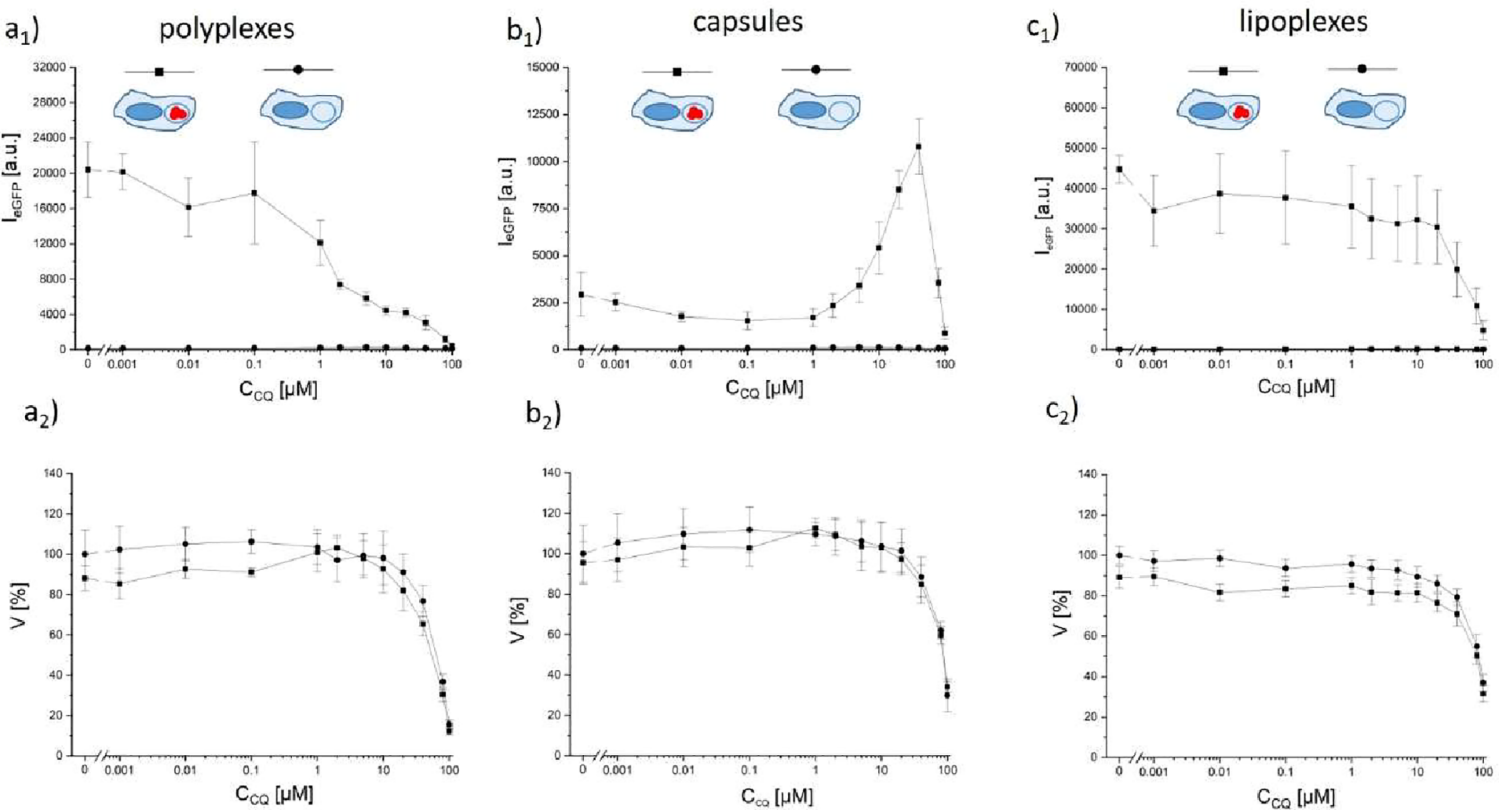
Transfection efficiency in terms of eGFP expression versus chloroquine concentration for (a) polyplexes, (b) capsules, and (c) lipoplexes.

## Conclusions

The experiments as demonstrated here are easy to carry out and should be easy to follow for other laboratories for other systems. With such experiments, basic facts, such as the influence of chloroquine on the uptake of particular matter by cells and transfection rates, can be conveniently probed, without having to go into molecular details. For such basic screening, standard NPs may be a good first model system for viruses. While we are fully aware that our chosen NP systems are not equal to the SARS-CoV-2 virus, they are still basic model systems resembling the particulate nature of the SARS-CoV-2 virus (while completely neglecting (bio-) molecular details). Data show that several generalized statements in the literature might, in fact, be true for a particular system but are not true in general. We have presented here counterexamples under which chloroquine increases NP uptake and transfection, which would be the opposite of the desired effect concerning preventing viral infection. Again, our data do not explicitly exclude other biochemical effects of chloroquine, which might be in favor of preventing viral infection. Our data are exclusively valid for the effect of the particulate nature of viruses, not for their biochemical properties. We want to point out, in particular, the toxic nature of chloroquine, which, of course, is well reported. The important fact is that in some cases, the effects of chloroquine on NP-based uptake and transfection start at the same concentration as when toxic effects come into play.

The next level of a more appropriate model system, which would come closer to the features of the SARS-CoV-2 virus, could involve dedicated surface functionalization, such as NP conjugation with spike proteins.^60^ By its nature, even such an improved model system will not reach the complexity of the “original”, i.e., SARS-CoV-2 viruses. The reason in the first place to use model systems is to reduce complexity to break down effects to key parameters. For this, NPs are a convenient platform.

## Methods

The synthesis and characterization of the TAMRA-labeled Au NPs were carried out according to standard protocols.^38^ Flow cytometry-based uptake studies and viability assays were done according to standard methods.^40,49^ Transfection studies were carried out according to previously published protocols.^54,55,58^ Details can be found in the Supporting Information.

## Supplementary Material



## Data Availability

All data are provided in the Supporting Information.

## Notes

Experimental procedures; raw data of the flow cytometry and microscopy experiments; fluorescence spectrum of the NPs (Figure S1) at different pH (Figure S3) and upon exposure to chloroquine (Figures S4–S6); microscopy image of the capsules (Figure S2); microscopy images of cells with endocytosed NPs (Figures S7, S8); flow cytomery data of cells with endocytosed NPs and controls (Figures S10, S11); recordings of the pH in endosomes/lysosomes (Figures S12–S15); flow cytometry data of GPF expression upon transfection (Figures S16–S21) (PDF)
