# Supplementary material for: Uptake of Nanoparticles as a Model System for Viruses under the Presence of Chloroquine
Source: ACS Nanosci Au. 2026 Jan 23;6(2):305–12. doi: 10.1021/acsnanoscienceau.5c00183 (PMC13087937; doi:10.1021/acsnanoscienceau.5c00183)
Supplement: Supplementary file 1 [file ng5c00183_si_001.pdf]

Supporting Information

Uptake of nanoparticles as model system for viruses under the presence of chloroquine

Gan Chen[1,2], Dingcheng Zhu[1], Yaofeng Zhao[1], Timothy K. Soh[3,4,5,6], Ruixia Wang[1,7], Neus Feliu[1], Wolfgang J. Parak[1*]

[1]Center for Hybrid Nanostructures (CHyN), Universität Hamburg, Hamburg, Germany

[2]Academy of Military Medical Sciences, Beijing, China

[3]Centre for Structural Systems Biology, Hamburg, Germany

[4]Hannover Medical School, Institute of Virology, Hannover, Germany

[5]Cluster of Excellence RESIST (EXC 2155), Hannover Medical School, Hannover, Germany

[6]Leibniz Institute of Virology (LIV), Hamburg, Germany

[7]Deutsches Elektronen-Synchrotron DESY, Hamburg, Germany

*corresponding author: wolfgang.parak@uni-hamburg.de

1. Reagents

2. Synthesis of materials

3. Effect of different pH or chloroquine concentrations on the fluorescence intensity of the Au nanoparticles

4. Cell culture and cytotoxicity studies

5. Cellular uptake of Au nanoparticles

6. Intracellular pH calibration curves for the pH-responsive capsules

7. Measurement of lysosomal pH at different chloroquine concentrations

8. Gene transfection quantification

9. References

1. Reagents

Chloroquine diphosphate salt (Chloroquine, #C6628), Gold(III) chloride trihydrate ($HAuCl_4 \cdot 3H_2O$, #520918), poly(allylamine hydrochloride) (PAH, M_w ~ 56 kDa, #283223), Poly(sodium 4-styrenesulfonate) (PSS, M_w ~ 70 kDa, #243051), Poly (isobutylene-alt-maleic anhydride) (M_w ~ 6 kDa, #531278), ascorbic acid (#255564), branched poly(ethyleneimine) (PEI, M_w ~ 25 kDa, #408727), potassium chloride (#P9541), magnesium sulfate (#M2643), citric acid (#251275), sodium carbonate (#S7795), calcium chloride dehydrate (#223506), ethylenediaminetetraacetic acid disodium salt (EDTA, #E26290), resazurin (#R7017), monensin sodium hydrate (#46468), 4-(2-hydroxyethyl)-1-piperazineethanesulfonic acid sodium salt (HEPES, #H3784), and sodium borohydride (#71320) were purchased from Sigma-Aldrich. Nigericin (#481990) was purchased from Millipore. Lipofectamine 2000 (#11668019) and dextran SNARF-1 (#2147784) were purchased from Invitrogen. Tetramethylrhodamine (TAMRA, #471C0) was purchased from Lumiprobe. Sodium chloride (NaCl, #HN00.2) was purchased from Carl Roth. Trypsin-EDTA (#25300-054), Dulbecco′s Modified Eagle′s Medium (DMEM, #41966), and penicillin-streptomycin solution (#15140122) were purchased from Thermo Fisher Scientific. Enhanced green fluorescent protein (eGFP)-encoding plasmid (peGFP) was kindly provided by the School of Medicine, Zhejiang University.[1] Milli-Q water was used for all experiments.

2. Synthesis of materials

2.1 Synthesis of fluorescence-labelled Au nanoparticles
2.2 Synthesis of pH-responsive microcapsules

2.1 Synthesis of fluorescence-labelled Au nanoparticles

Spherical gold nanoparticles (Au NPs), with around d_c = 4 nm core diameter, were synthesized following an existing standard procedure, in which they are embedded in the amphiphilic polymer poly(isobutylene–alt–maleic anhydride)–graft–dodecyl (PMA).[2, 3] The polymer hereby was labelled with the fluorophore TAMRA. TAMRA-labeled PMA was synthesized following an existing synthesis procedure.[3, 4] Briefly, 113.4 mg PMA was dissolved in 20 mL N, N-dimethylformamide (DMF), followed by sonication. Subsequently, 12.5 mg TAMRA, 102.4 mg dodecylamine, and 2.0 mL triethylamine (TEA) were added to the solution. The reaction mixture was stirred at 75 °C in the dark overnight for conjugation. Then, the solvent was evaporated, and the TAMRA-labeled PMA was dissolved in chloroform. The dye-modified polymer was used for coating of the Au NPs without further purification, as reported previously.[3, 5] A fluorescence spectrum of the TAMRA labelled Au NPs (Au-PMA-TAMRA NPs) is shown in Figure S1.

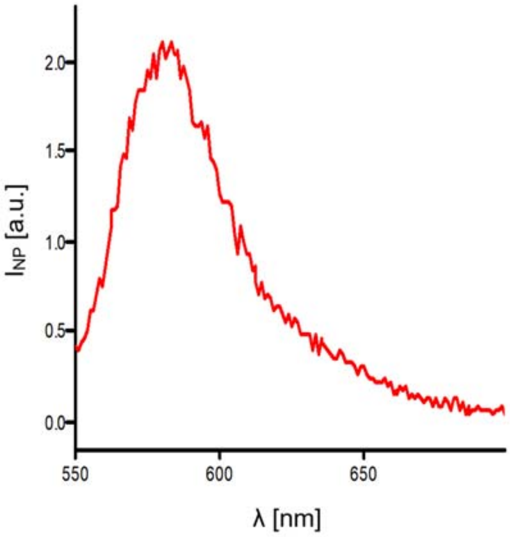

Figure S1. Fluorescence emission spectrum of Au-PMA-TAMRA NPs dissolved in water (excitation wavelength λ_{ex} = 488 nm, detected emission wavelength range λ_{em} = 550–700 nm).

2.2 Synthesis of pH-responsive microcapsules

pH-responsive capsules were fabricated using layer-by-layer assembly of PAH and PSS onto a SNARF-loaded spherical calcium carbonate template by implementing existing methods.[6, 7] Briefly, 0.615 mL of Na_2CO_3 (0.33 M) solution was mixed with 500 µL of SNARF-dextran (1 mg/mL) solution. After stirring for 5 min, the mixture was added into 0.615 mL of 0.33 M $CaCl_2$ under stirring (1000 rpm). Stirring was continued for 30 s, followed by another 2 min incubation time without stirring. The SNARF-loaded $CaCO_3$ particles were washed twice using ultrapure water, then alternating layers of negatively charged PSS (2 mg/mL) and positively charged PAH (2 mg/mL) were deposited onto the SNARF-loaded $CaCO_3$ particles. The obtained template cores overcoated with four bilayers of (PAH/PSS) were immersed in 1 mL EDTA solution (0.1 M) at 4 °C overnight to remove the $CaCO_3$ templates, leaving the SNARF inside the remaining polymer shell, thus forming SNARF-loaded capsules. After washing them three times with ultrapure water, the pH-responsive capsules were stored at 4 °C until further use. Figure S2 shows a microscopy image of the capsules. For further characterization procedures we refer to previous studies.[7, 8]

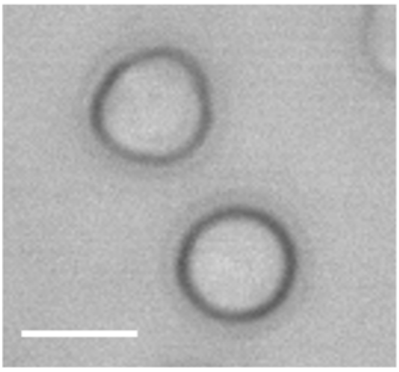

Figure S2. Bright-field microscopy image of SNARF-loaded pH-responsive capsules. The scale bar represents 5 µm.

3. Effect of different pH or chloroquine concentrations on the fluorescence intensity of the Au nanoparticles

3.1 Effect of pH

3.2 Effect of different chloroquine concentrations

3.1 Effect of different pH

To detect the effect of pH on the fluorescence intensity of Au-PMA-TARMA NPs, phosphate-citric acid buffers (pH range 5.0-7.0) were prepared with different ratios of 0.2 M dibasic sodium phosphate and 0.1 M citric acid solution.[7] Both buffer solutions also contained 150 mM sodium chloride, 4 mM potassium chloride, and 1 mM magnesium sulfate. A 15 µL Au-PMA-TRAMA NP solution ($c_{NP} \approx 5$ µM) was added to 500 µL solutions of different pH (5.0, 5.4, 6.0, 6.4, and 7.0), and the fluorescence intensities of the NPs were detected using a fluorophotometer (Agilent Technologies) with an excitation wavelength λ_{ex} = 488 nm and emission recorded at wavelengths λ_{em} from 550-700 nm, see Figure S3. The fluorescence intensity I_{NP} at λ_{em} = 580 nm *versus* the pH was then plotted, see Figure 1b$_1$.

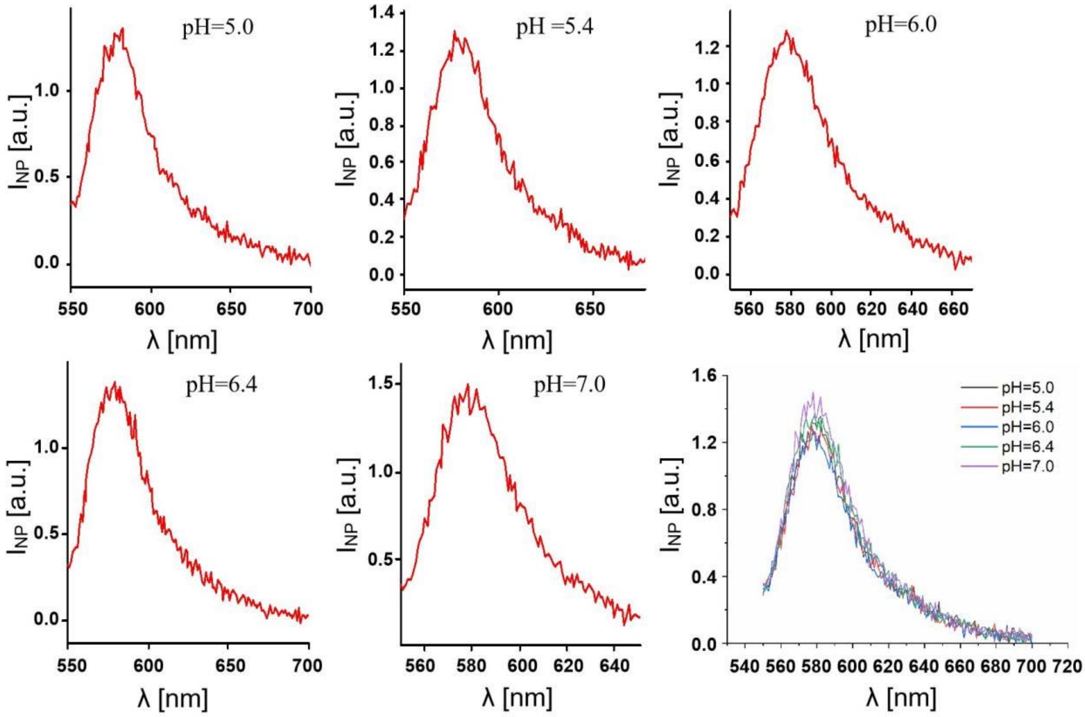

Figure S3. Fluorescence emission spectra of Au-PMA-TAMRA NPs immersed in buffers of different pH. The last image is an overlay of all spectra.

3.2 Effect of different chloroquine concentrations

Solutions with different chloroquine concentrations c_{CQ} were prepared by diluting the chloroquine stock solution with water as solvent. Then, 15 µL of Au-PMA-TRAMA NP solution ($c_{NP} \approx 5$ µM) was added to 500 µL solutions of the different chloroquine concentrations c_{CQ} (0, 0.001, 0.01, 0.1, 1, 2, 5, 10, 20, 40, 80, and 100 µM). Fluorescence intensities I_{NP} were detected using a fluorophotometer with excitation wavelength (λ_{ex}) of 488 nm and the emission was recorded in the range of 550-700 nm, see Figure S4. An overlay of all curves shown in Figure S4 is shown in Figure S5. As control the fluorescence of the different chloroquine solutions (without added NPs) was measured, see Figure S6. Data indicate that the chloroquine fluorescence is much lower that the NP fluorescence. The fluorescence intensity at λ_{em} = 580 nm *versus* chloroquine the chloroquine concentrations for plain chloroquine and chloroquine + Au NP solutions is plotted in Figure 1b2.

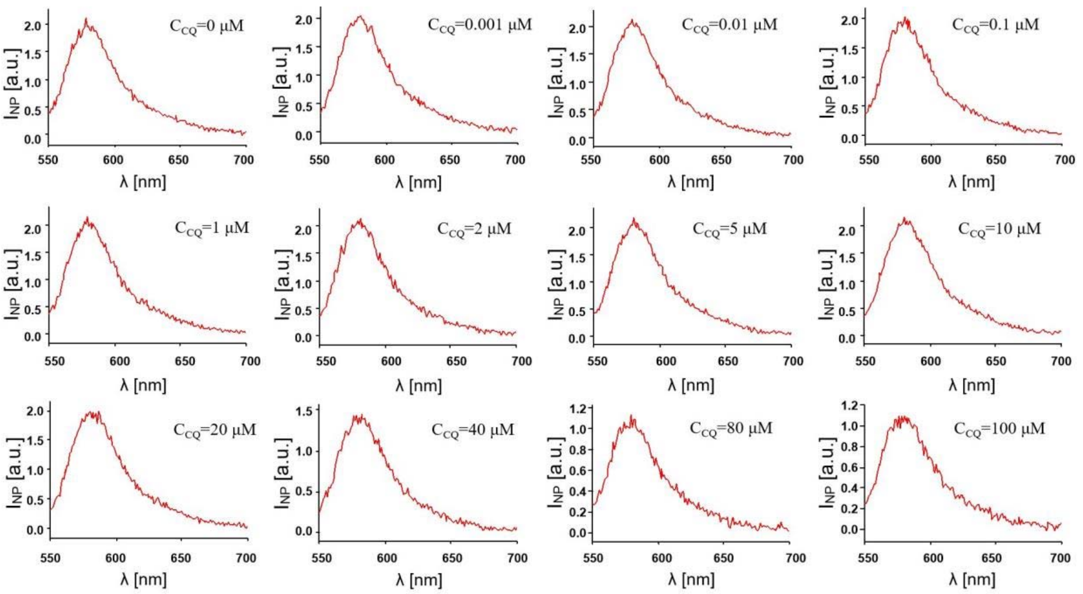

Figure S4. Fluorescence emission spectra $I_{NP}(\lambda)$ of Au-PMA-TAMRA NPs immersed in buffers with different chloroquine concentrations c_{CQ}.

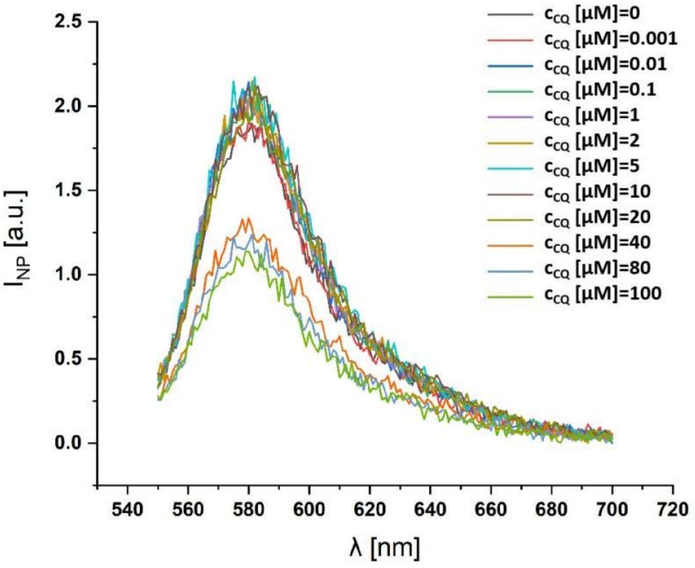

Figure S5. Overlay of the fluorescence spectra shown in Figure S4.

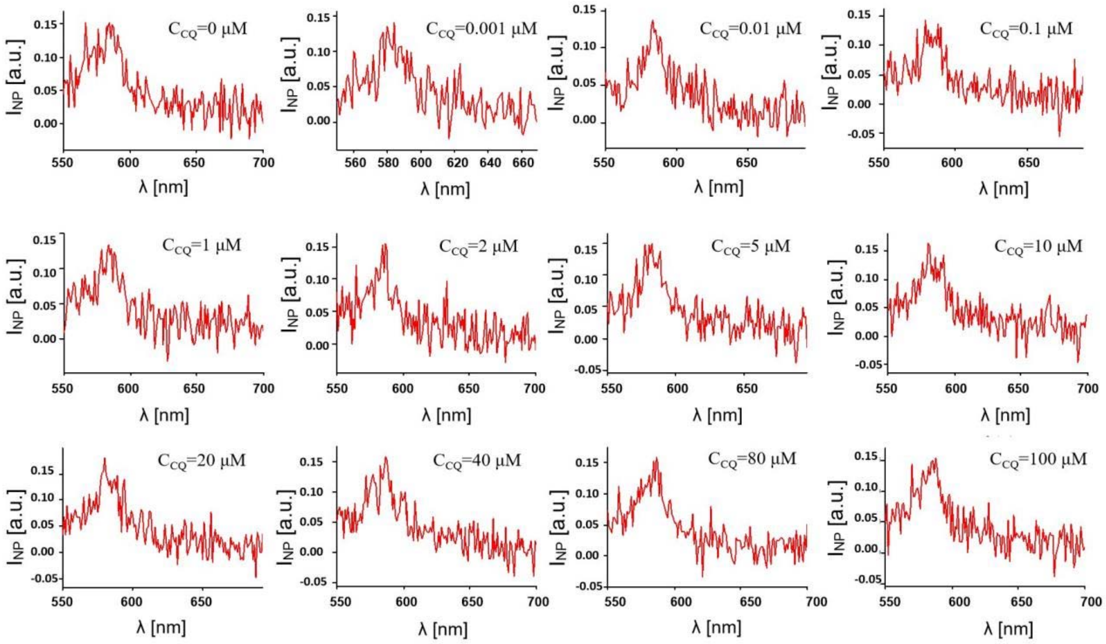

Figure S6. Fluorescence emission spectra $I_{NP}(\lambda)$ of buffers with different chloroquine concentrations c_{CQ} without added NPs.

4. Cell culture and cytotoxicity studies

4.1 Cell culture
4.2 Cytotoxicity studies

4.1 Cell culture

HeLa cells were cultured in high-glucose DMEM supplemented with 1% penicillin-streptomycin solution and 10% fetal bovine serum (FBS; Biochrom) in an incubator (37 °C, 5% CO_2). The cells were passaged by trypsinization with trypsin-EDTA solution and seeded in culture plates 24 h before cellular experiments.

4.2 Cytotoxicity studies

Cytotoxicity studies were performed using a resazurin-based assay.[3] HeLa cells were seeded in 48-well culture plates (Corning Costar, 0.95 cm^2 seeding area *per* well, 0.25 mL medium *per* well), with 1.5×10^4 cells *per* well, 24 h before experiments. HeLa cells were exposed to different NP/chloroquine concentrations (final NP and chloroquine concentrations of the medium: c_{NP} and c_{CQ}, respectively). Then, after incubation for 24 h, the cell culture medium was replaced by 0.8 mL of fresh complete culture medium with resazurin solution (0.025 mg/mL). Thereafter, the cells were incubated for 3 h at 37 °C, 5% CO_2. Finally, the fluorescence intensities of the cell culture supernatant were measured using a fluorimeter with an excitation wavelength (λ_{ex}) of 550 nm at the emission wavelength (λ_{em}) of 600 nm. The emission intensity correlates to cell viability V. The emission intensity at c_{CQ} = 0 was normalized to a cell viability V = 100%.[3] The viability *versus* chloroquine concentration is plotted in Figure 1a_2.

5. Cellular uptake of Au nanoparticles

5.1. Confocal microscopy
5.2. Flow cytometry

5.1. Confocal microscopy

50,000 HeLa cells were suspended in 2 mL of serum supplemented DMEM cell medium (contained chloroquine at different concentrations c_{CQ} and NPs with a concentration of $c_{NP} \approx$ 60-65 nM). After mixing, the cell solution was added into 2.5 cm diameter petri dishes and cultured at 5% CO_2 and 37 °C for 24 h. The cell sample was then washed with 0.01 M PBS three times and then nuclei were stained by Hoechst 33342, the cell membrane with WGA@Alexa fluor 488, and endosomes/lysosomes wiht lysotracker green DND-26. The staining protocols were taken form the ThermoFisher website and are similar to previous reports.[3, 9]

Z-stack imaging was performed with a two-photon microscope (ZEISS LSM 880 with Airyscan) with the following settings for the respective channels: Hoechst 33342: λ_{ex} = 405 nm, λ_{em} = 410-496 nm; WGA@Alexa fluor 488: λ_{ex} = 488 nm, λ_{em} = 496-550 nm; Lysotracker green DND-26: λ_{ex} = 488 nm, λ_{em} = 553-606 nm; Au NPs: λ_{ex} = 633 nm, λ_{em} = 647-759 nm. The distance between the different z-stack images is 0.6 µm.

For every concentration of chloroquine 3 sets of images were recorder (#1, #2, #3, see Figure S7). We note that increase in the chloroquine concentrations slightly increased the fluorescence intensity of the lysotracker labelling and the internalized NPs. For high chloroquine concentrations of c_{CQ} = 100 µM most cells were found dead, and thus no z-stacks were recorded.

c_{CQ} = 0 μM (#1)

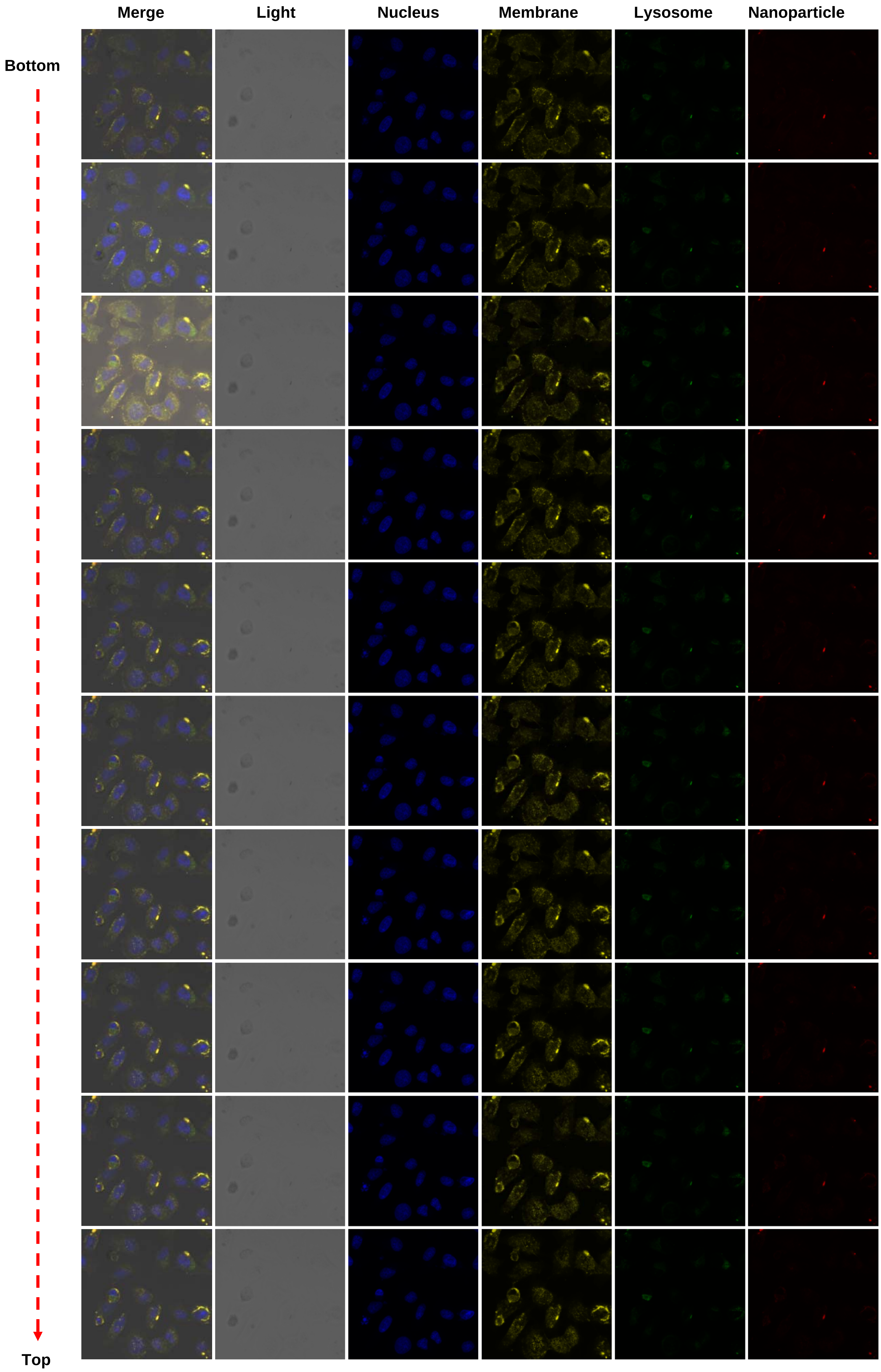
Merge
Light
Nucleus
Membrane
Lysosome
Nanoparticle
Bottom
Top

$c_{CQ} = 0\ \mu M$ (#2)

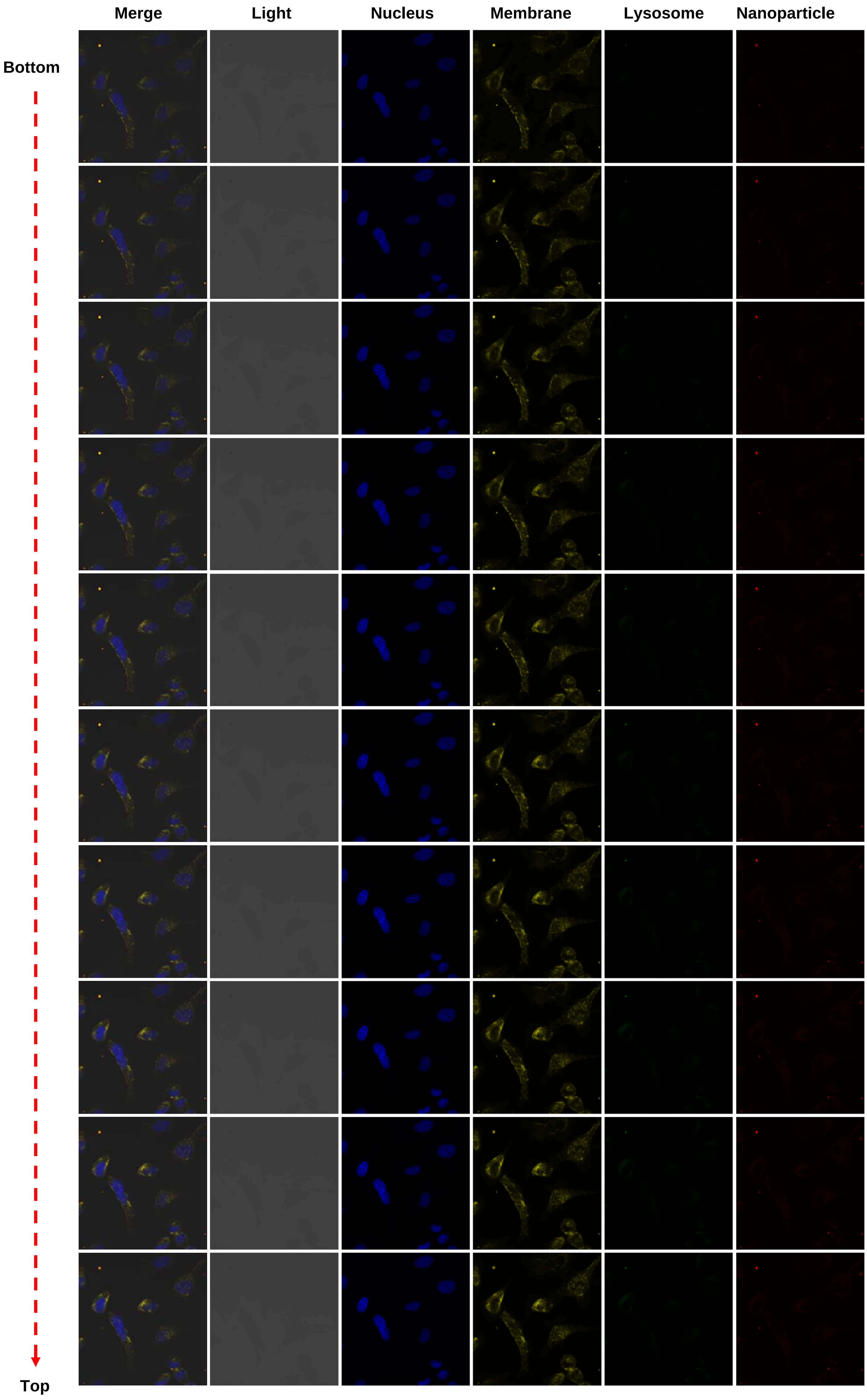
Merge
Light
Nucleus
Membrane
Lysosome
Nanoparticle
Bottom
Top

c_{CQ} = 0 µM (#3)

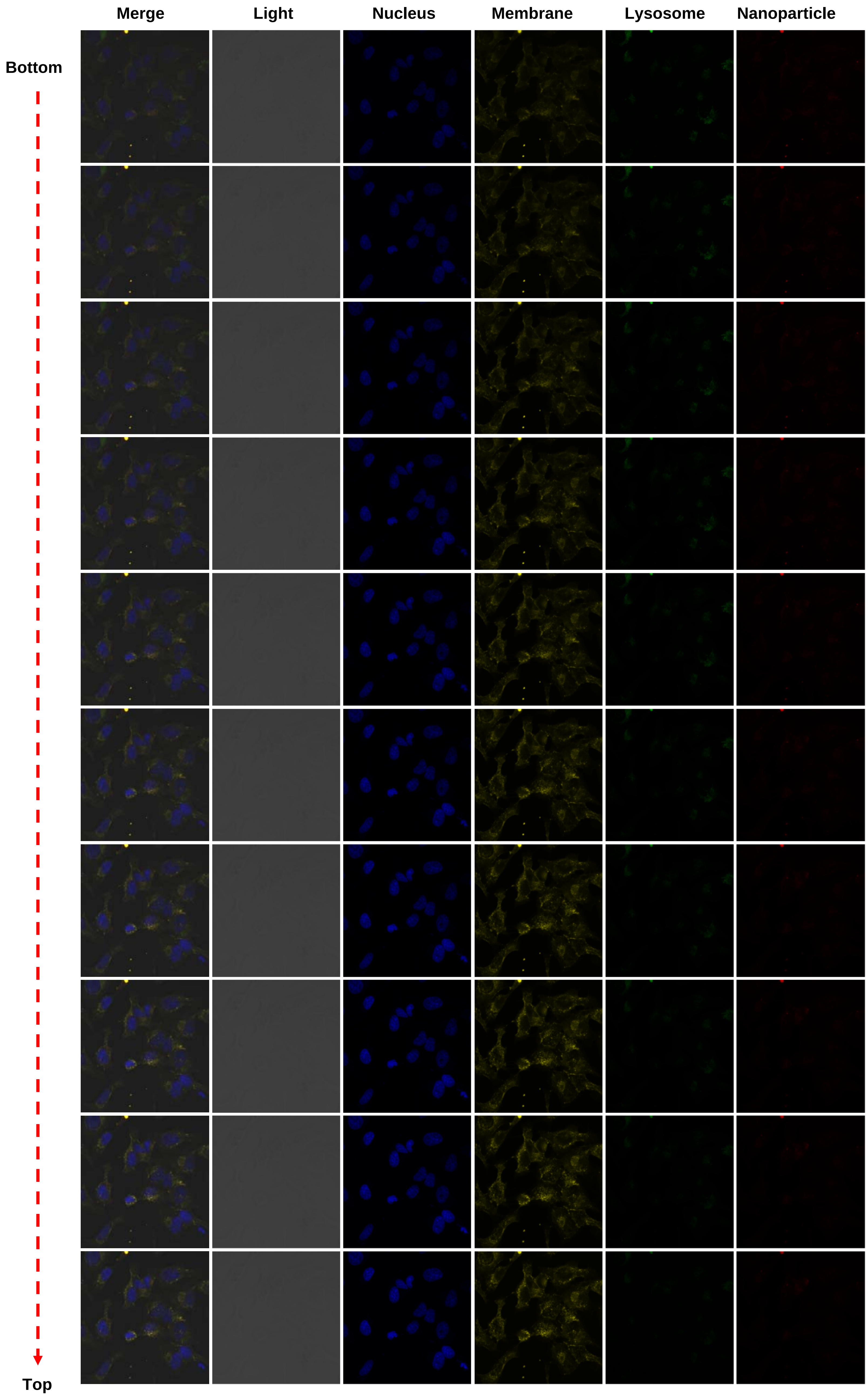
Merge
Light
Nucleus
Membrane
Lysosome
Nanoparticle
Bottom
Top

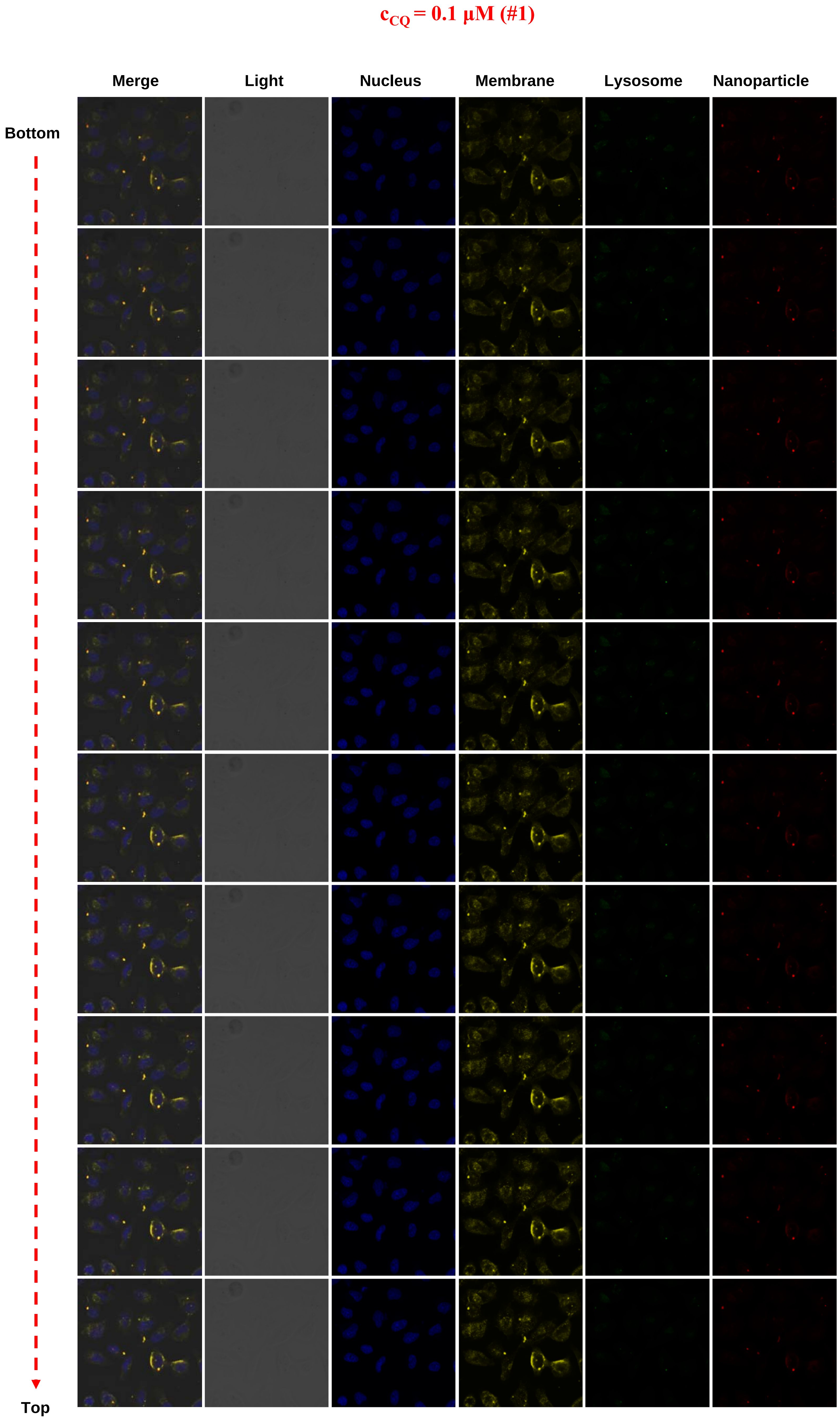
c_{CQ} = 0.1 μM (#1)
Merge
Light
Nucleus
Membrane
Lysosome
Nanoparticle
Bottom
Top

$c_{CQ} = 0.1\ \mu M$ (#2)

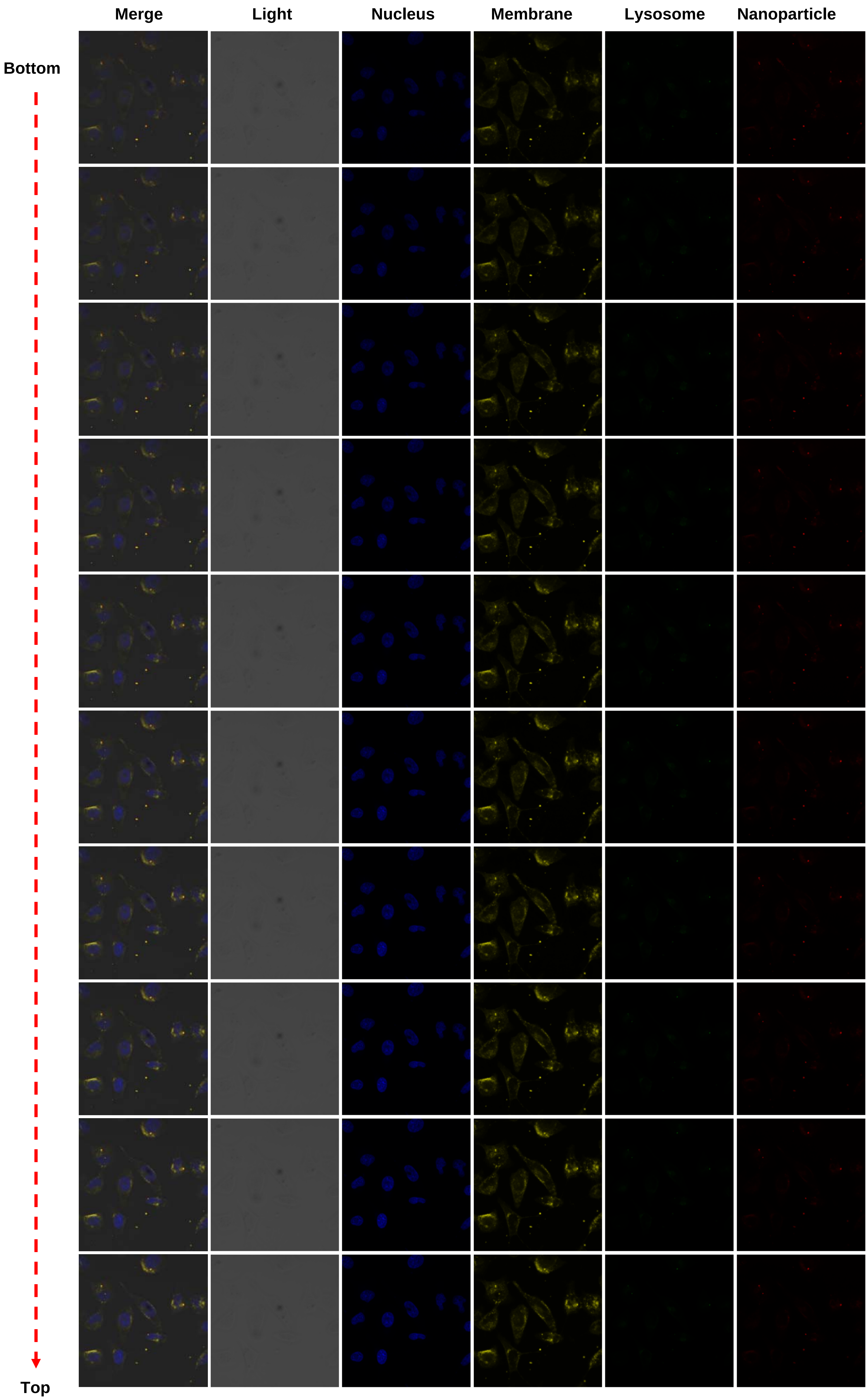
Merge
Light
Nucleus
Membrane
Lysosome
Nanoparticle
Bottom
Top

c_{CQ} = 0.1 μM (#3)

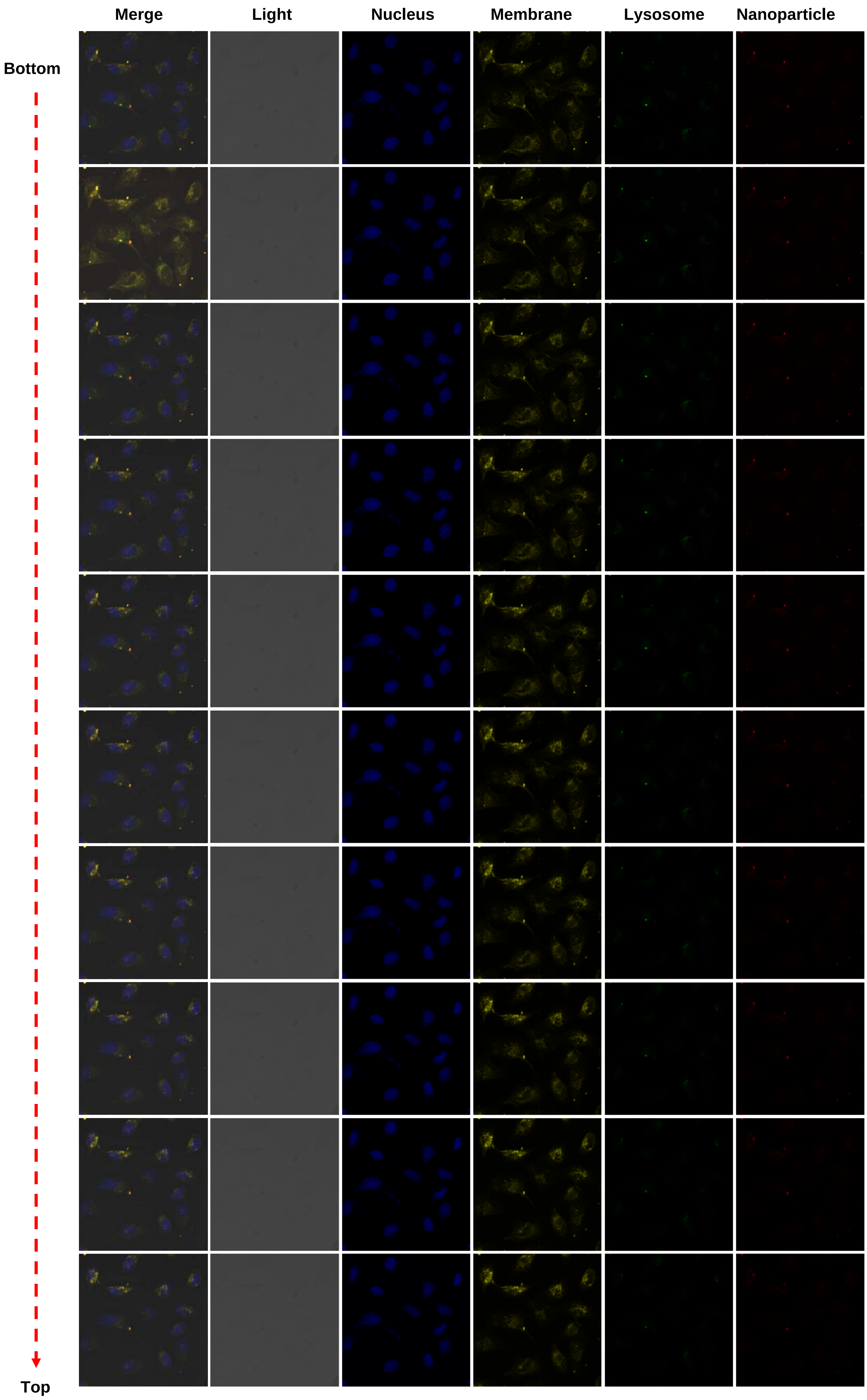

Merge
Light
Nucleus
Membrane
Lysosome
Nanoparticle
Bottom
Top

c_{CQ} = 1 µM (#1)

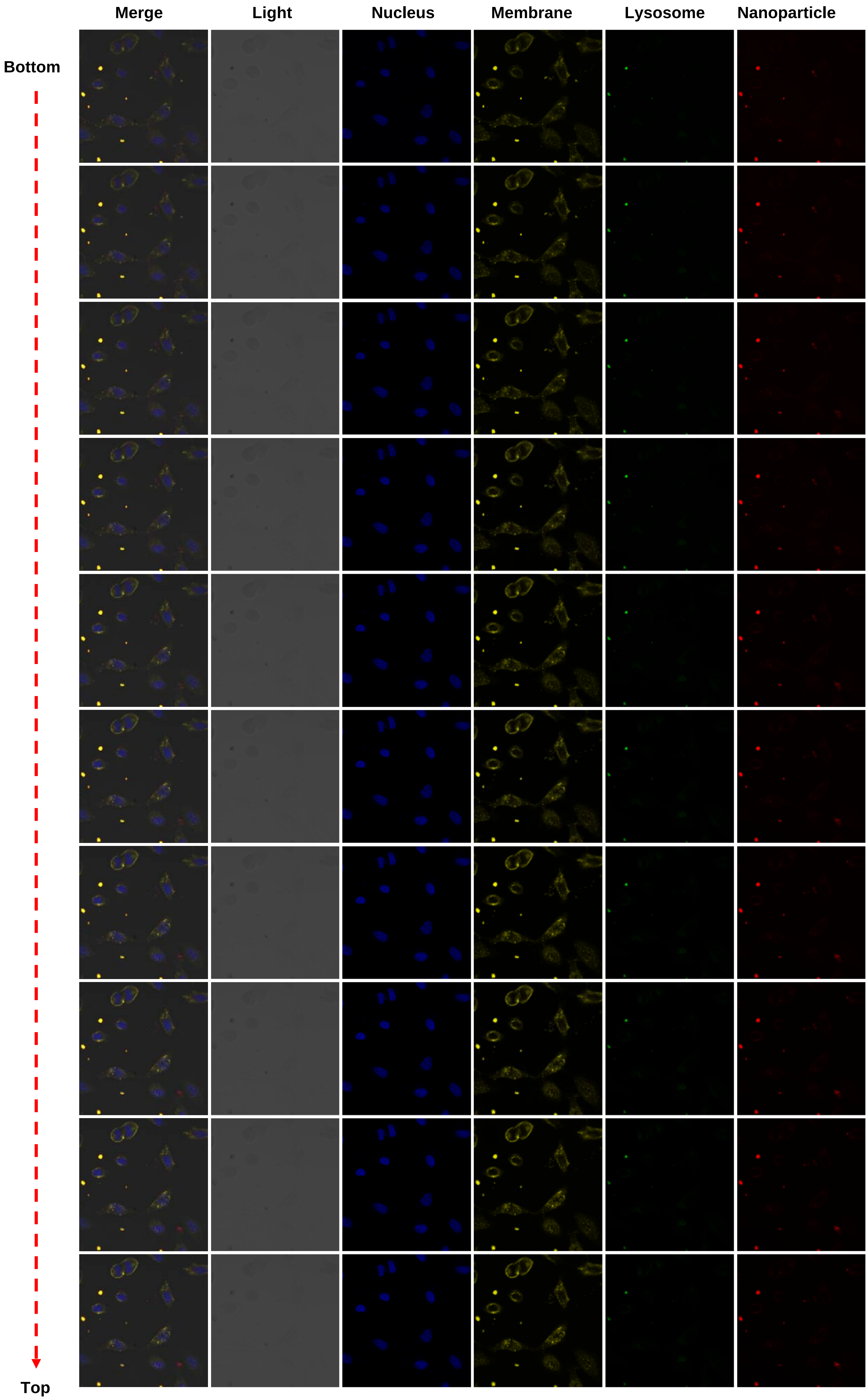
Merge
Light
Nucleus
Membrane
Lysosome
Nanoparticle
Bottom
Top

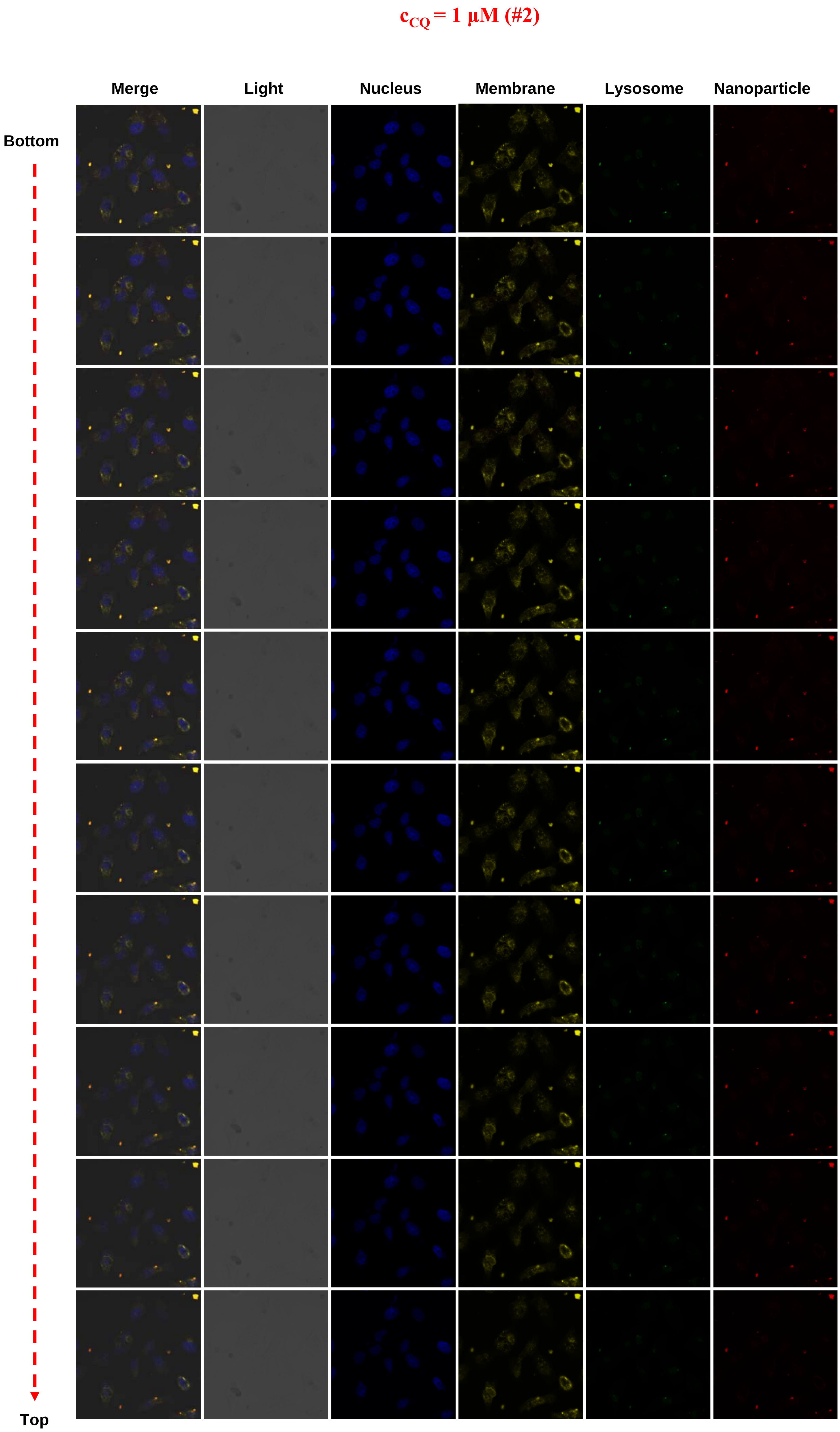
c_{CQ} = 1 μM (#2)
Merge
Light
Nucleus
Membrane
Lysosome
Nanoparticle
Bottom
Top

$c_{CQ} = 1\ \mu M$ (#3)

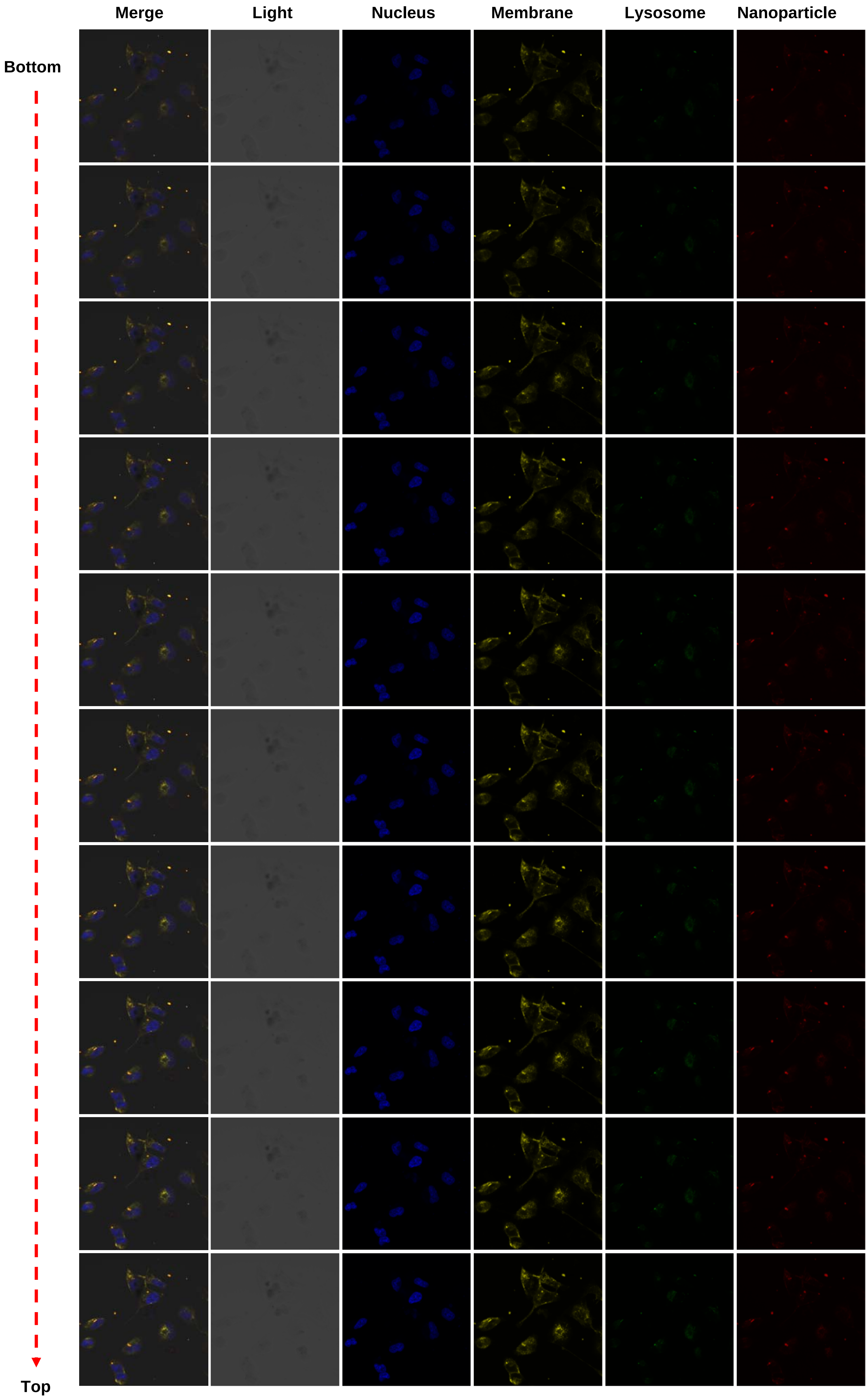
Merge
Light
Nucleus
Membrane
Lysosome
Nanoparticle
Bottom
Top

c_{CQ} = 10 μM (#1)

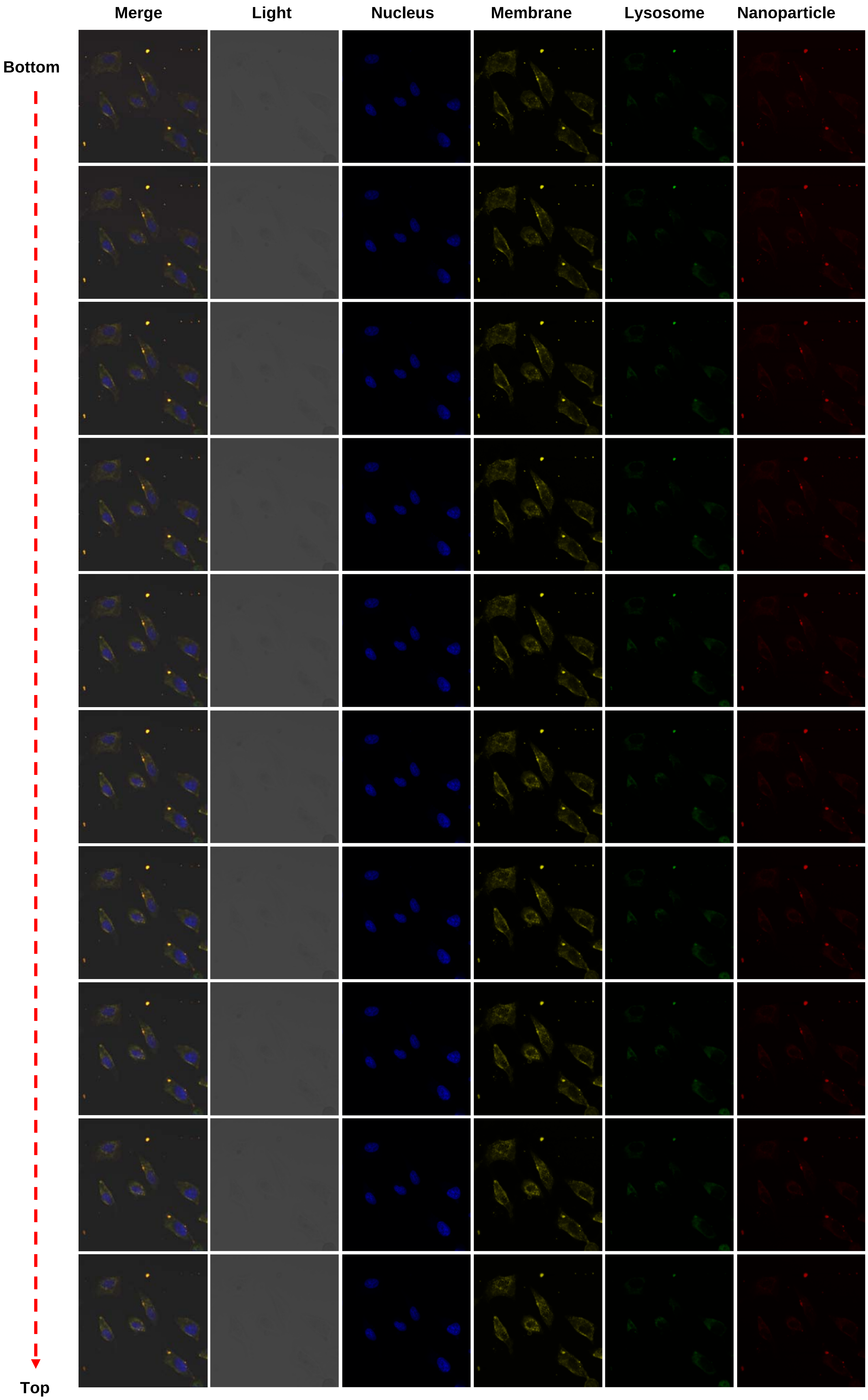
Merge
Light
Nucleus
Membrane
Lysosome
Nanoparticle
Bottom
Top

c_{CQ} = 10 μM (#2)

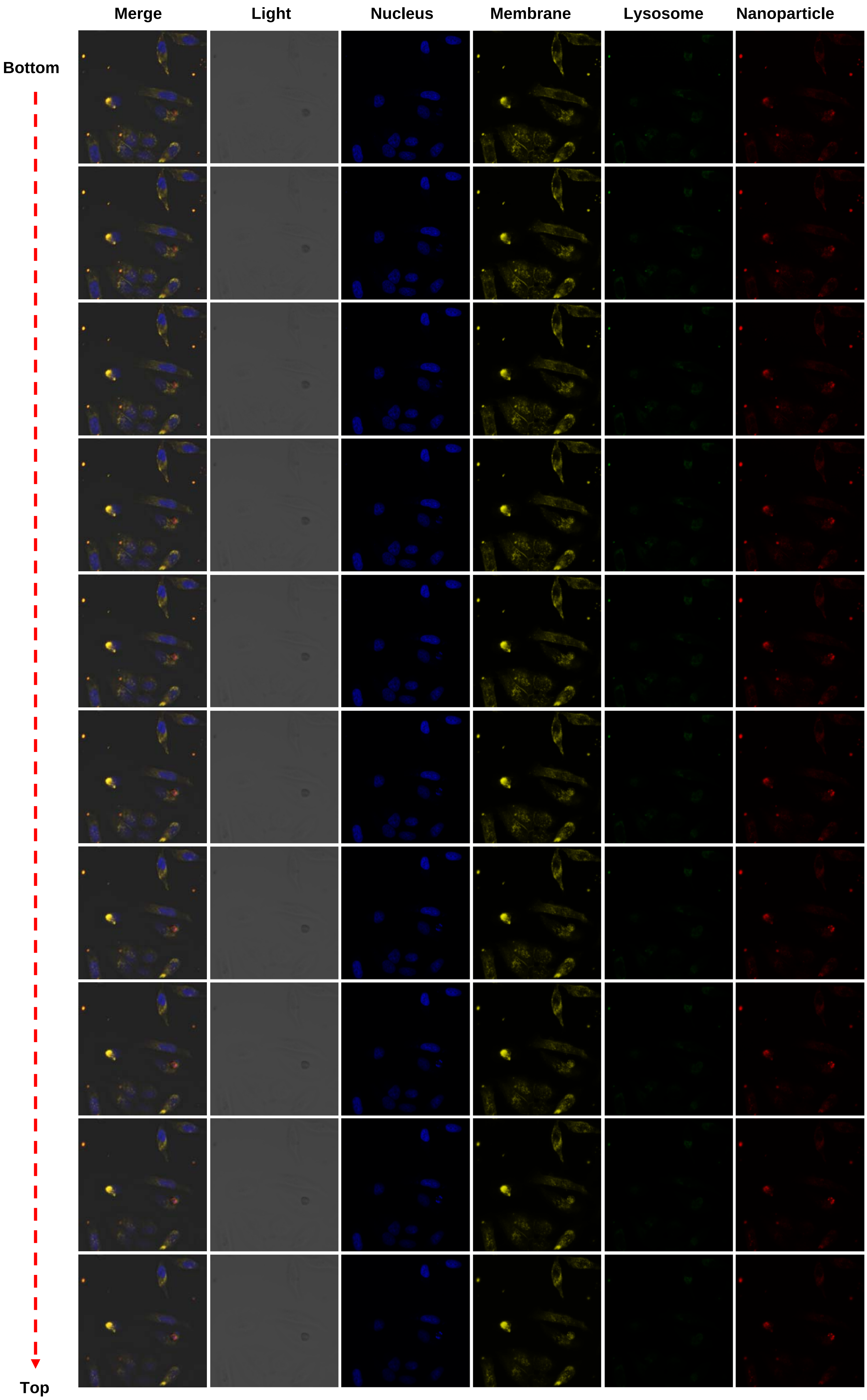
Merge
Light
Nucleus
Membrane
Lysosome
Nanoparticle
Bottom
Top

c_{CQ} = 10 μM (#3)

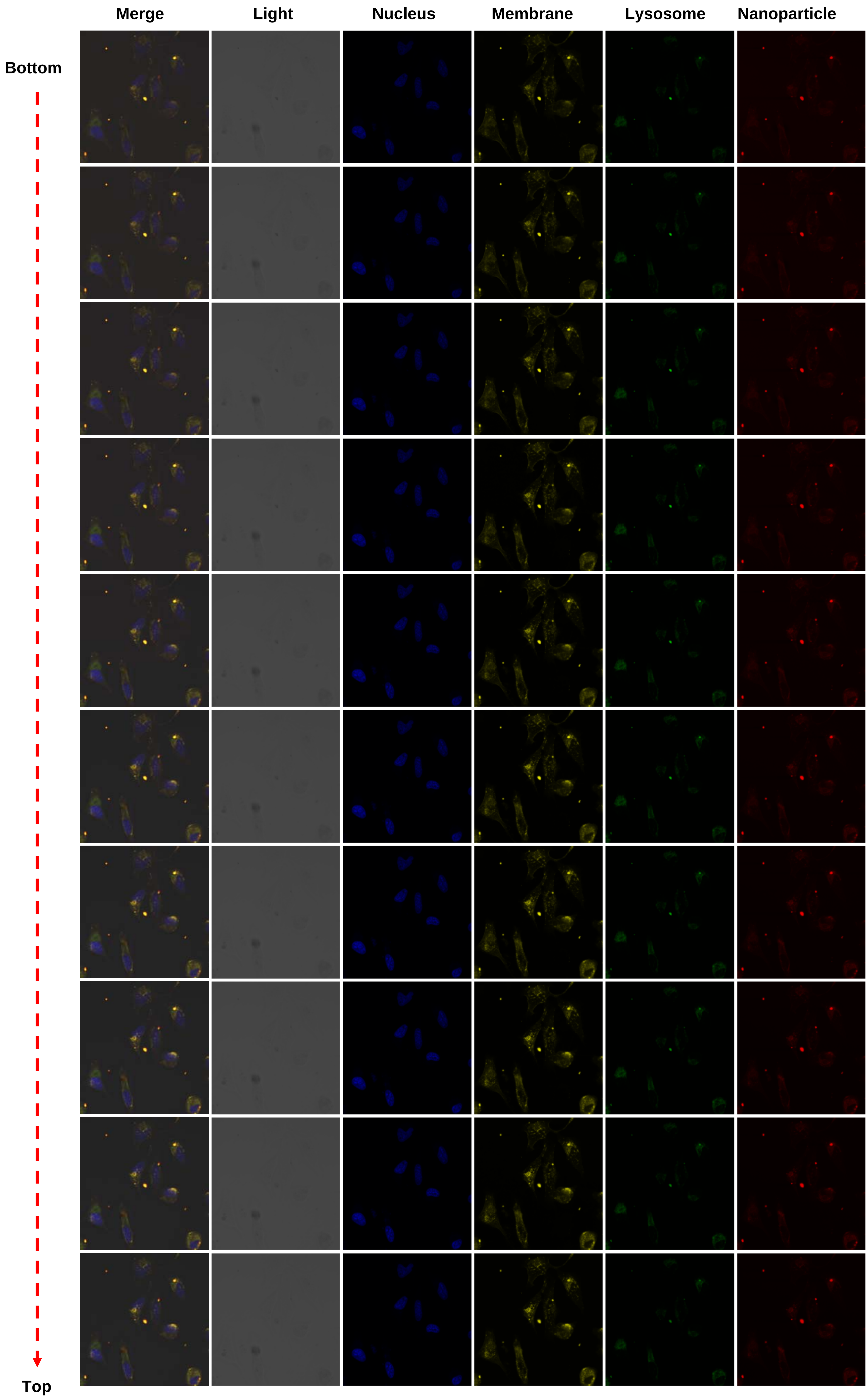
Merge
Light
Nucleus
Membrane
Lysosome
Nanoparticle
Bottom
Top

$c_{CQ} = 50\ \mu M$ (#1)

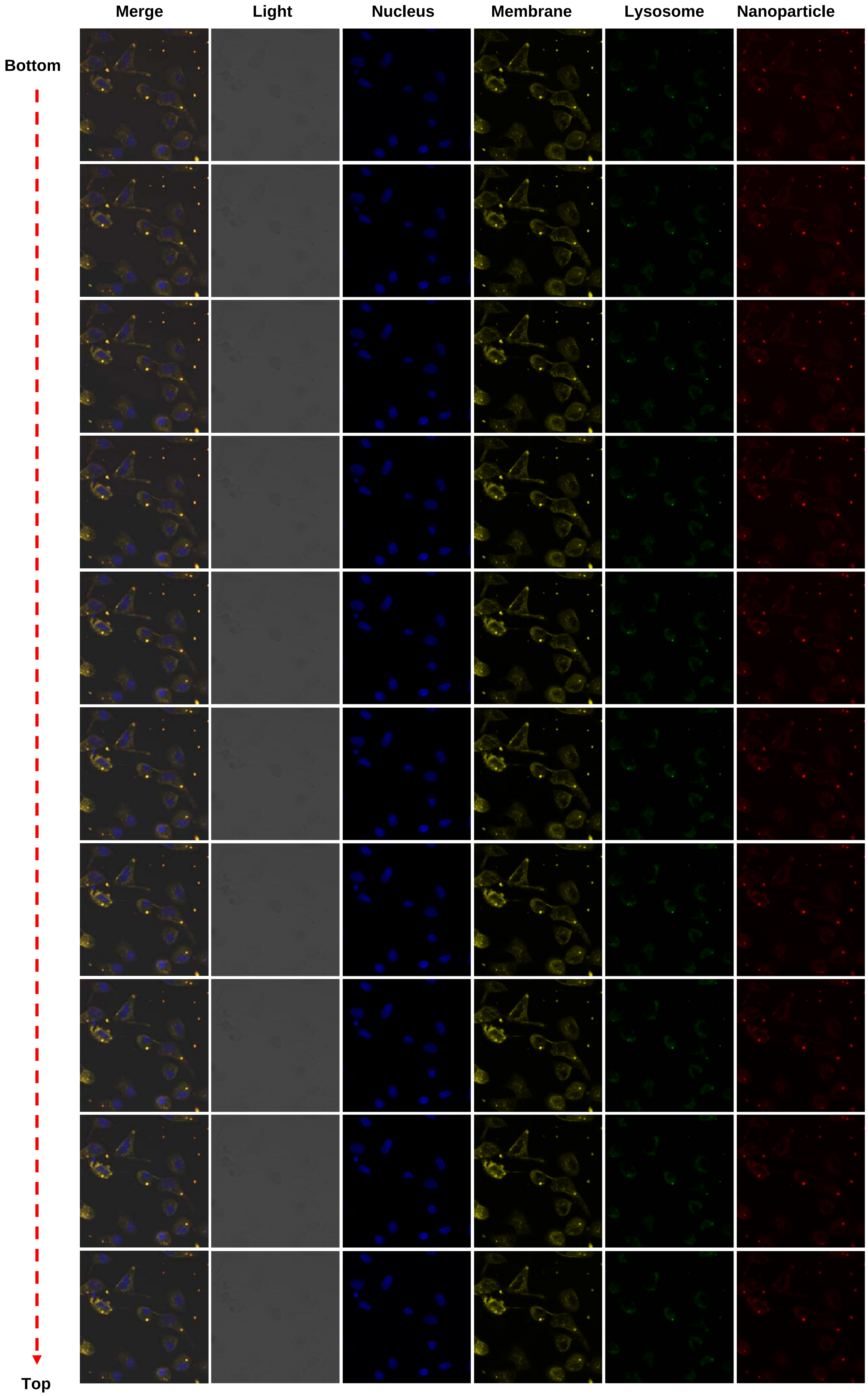
Merge
Light
Nucleus
Membrane
Lysosome
Nanoparticle
Bottom
Top

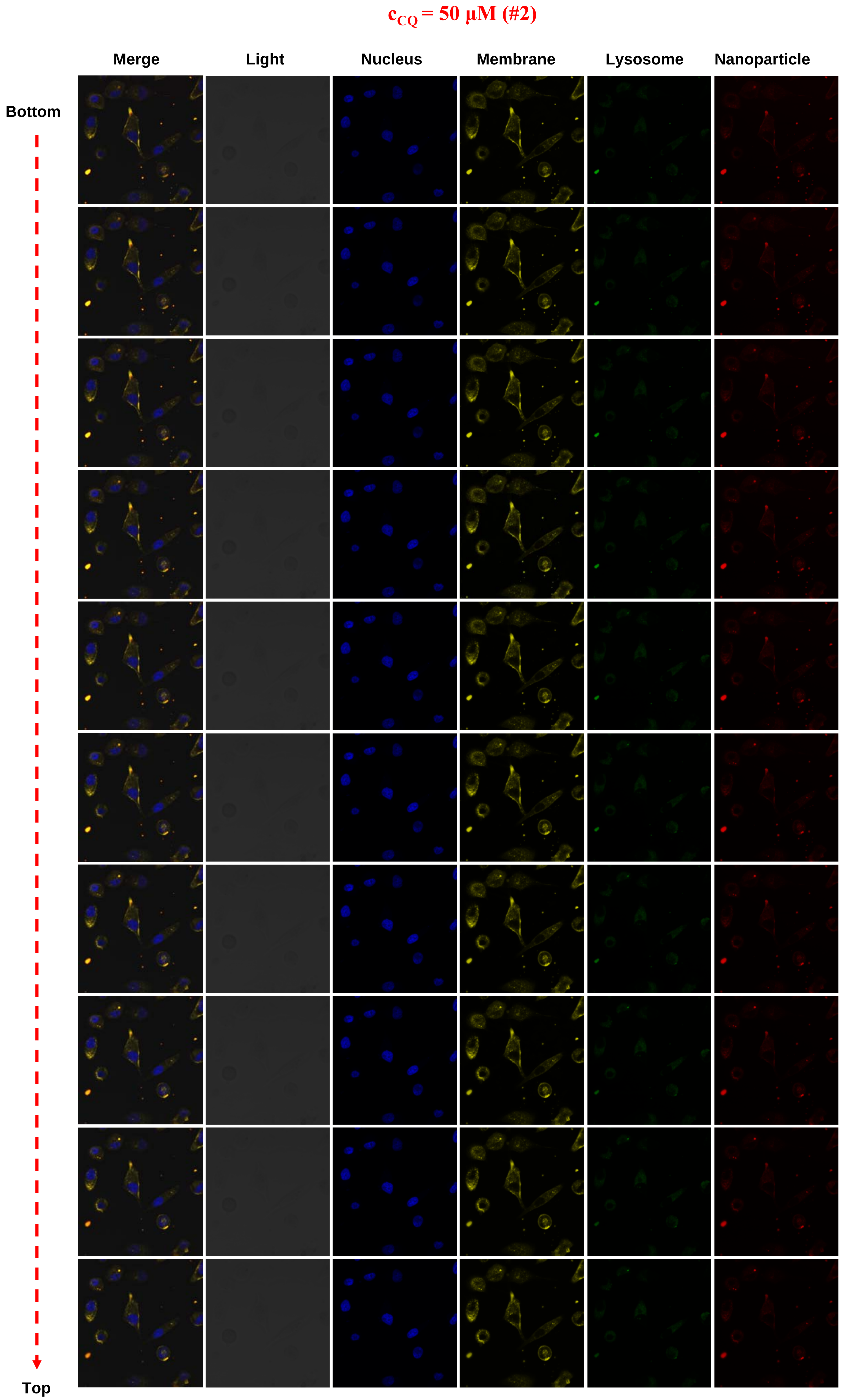

c_{CQ} = 50 µM (#2)
Merge
Light
Nucleus
Membrane
Lysosome
Nanoparticle
Bottom
Top

c_{CQ} = 50 μM (#3)

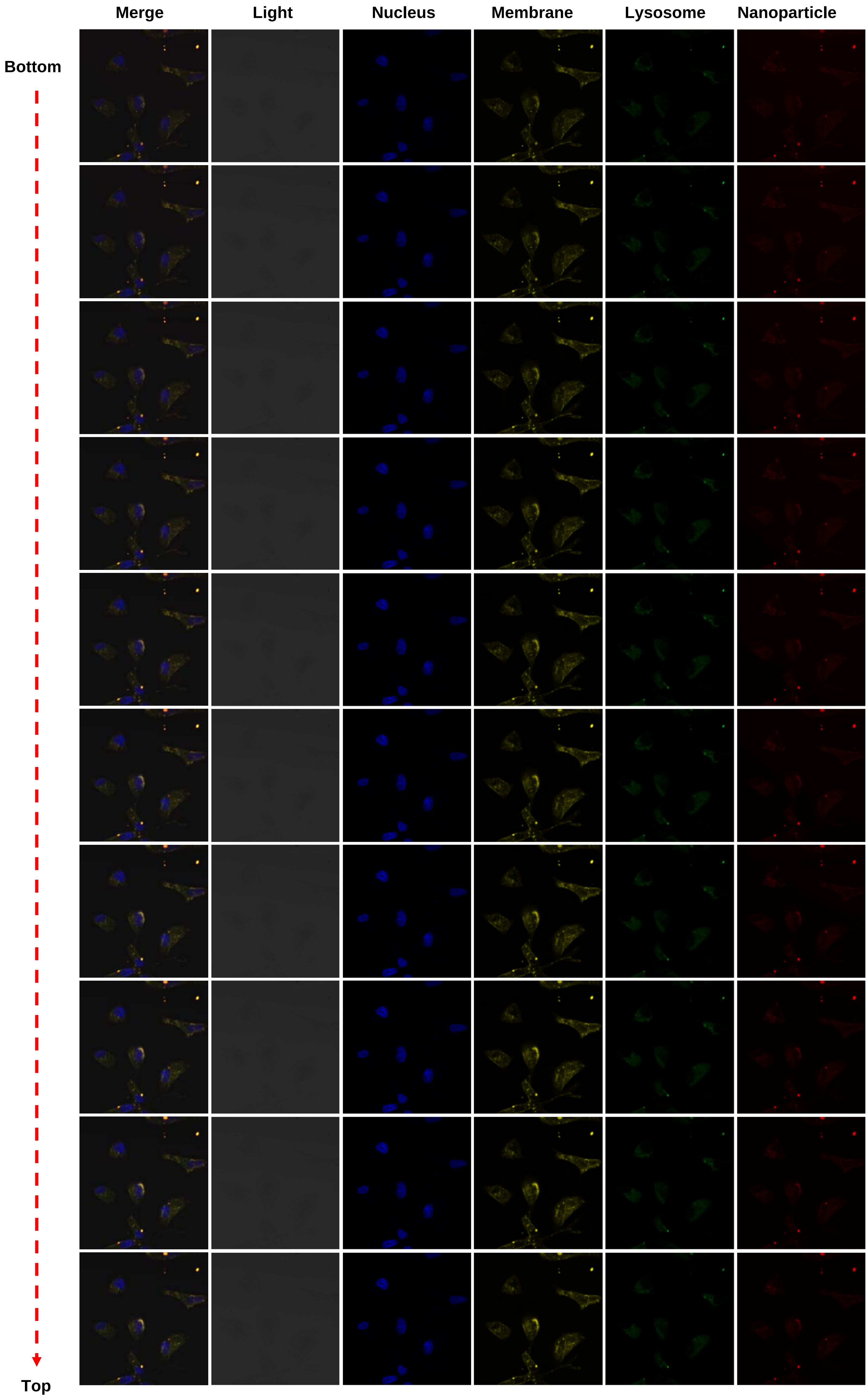
Merge
Light
Nucleus
Membrane
Lysosome
Nanoparticle
Bottom
Top

$c_{CQ} = 100$ µM (#1)

Light | Nucleus | Membrane | Lysosome | Nanoparticle

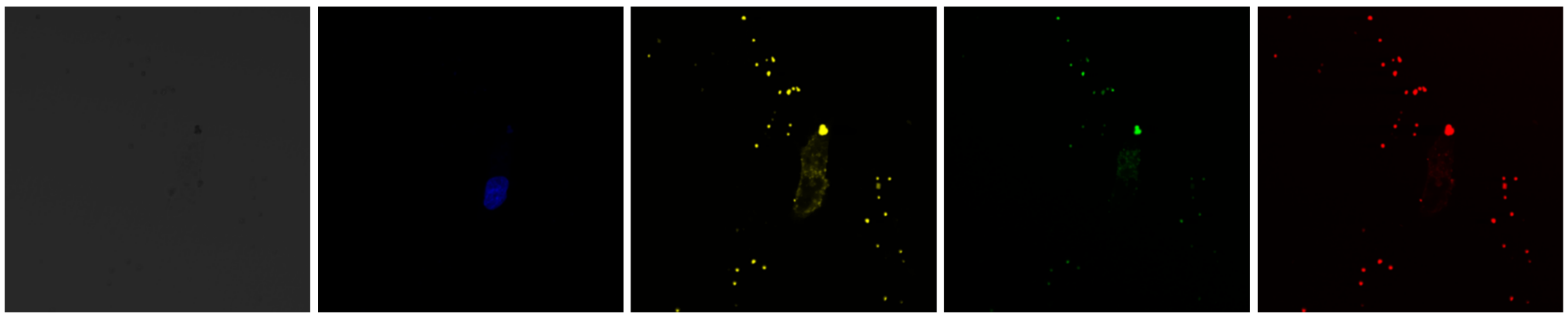

$c_{CQ} = 100$ µM (#2)

Light | Nucleus | Membrane | Lysosome | Nanoparticle

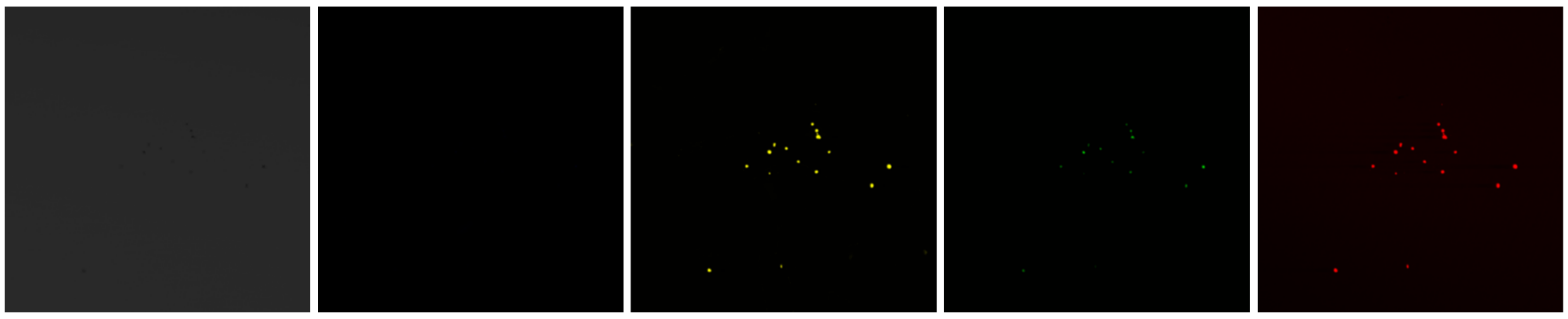

$c_{CQ} = 100$ µM (#3)

Light | Nucleus | Membrane | Lysosome | Nanoparticle

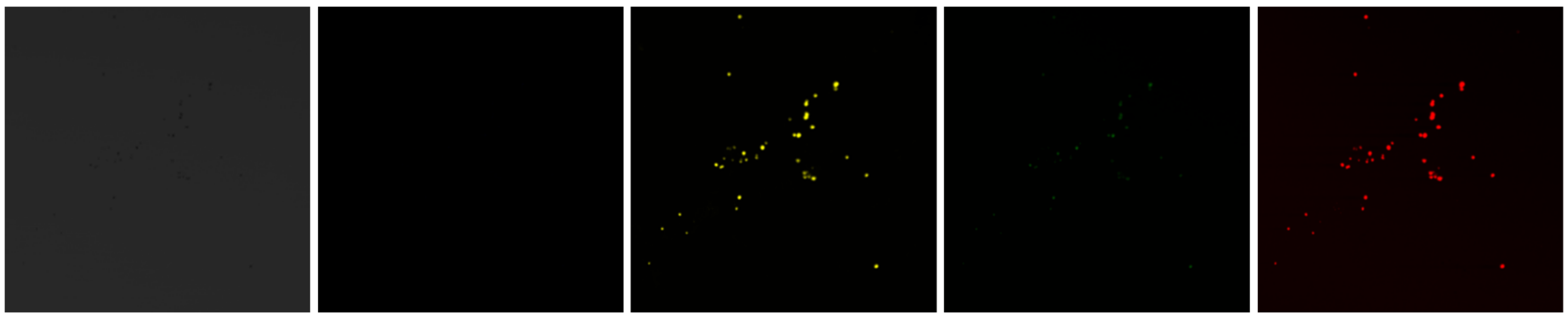

Figure S7. z-stack images of cells after incubation with chloroquine and NPs, showing the different fluorescence channels, the bright field channel, and the overlay of all channels.

5.2. Flow cytometry

HeLa cells were seeded in 24-well culture plates at 3.0×10^4 cells *per* well. After 24 h incubation at 37 °C and 5% CO_2, the cells were exposed to complete culture medium with different concentrations of chloroquine c_{CQ} (0, 0.001, 0.01, 0.1, 1, 2, 5, 10, 20, 40, 80, and 100 µM). Two hours later, the medium (0.5 mL) was replaced by complete culture medium with different concentrations of chloroquine c_{CQ} (0, 0.001, 0.01, 0.1, 1, 2, 5, 10, 20, 40, 80, and 100 µM) with or without the presence of Au-PMA-TAMRA NPs ($c_{NP} \approx 63$ nM). Confocal microscopy images confirmed endocytosis of the NPs, leading to the typical grainy pattern of NPs distributed in intracellular vesicles, see Figure S8. After 24 h incubation, the cells were washed twice using PBS, trypsinized, and then after trypsin neutralization and centrifugation the HeLa cells were resuspended in PBS. The fluorescence intensities of the cells (originating from the internalized fluorescent NPs) I_{NP} were measured using a FACScaliber flow cytometer (BD BioSciences, λ_{ex} = 488 nm, emission collected with a bandpass filter 586 ± 15 nm; corresponding to the FITC filter settings of the flow cytometer). The gating strategy for identifying events of single Hela cells, which had not been exposed to NPs, is shown in Figure S9, and the results for Hela cells after exposure to NPs are shown in Figure S10 and Figure S11 using the gating strategy from Figure S9. For each sample a minimum of 10000 cells was analyzed. The resulting uptake curves are plotted in Figure 1a_1.

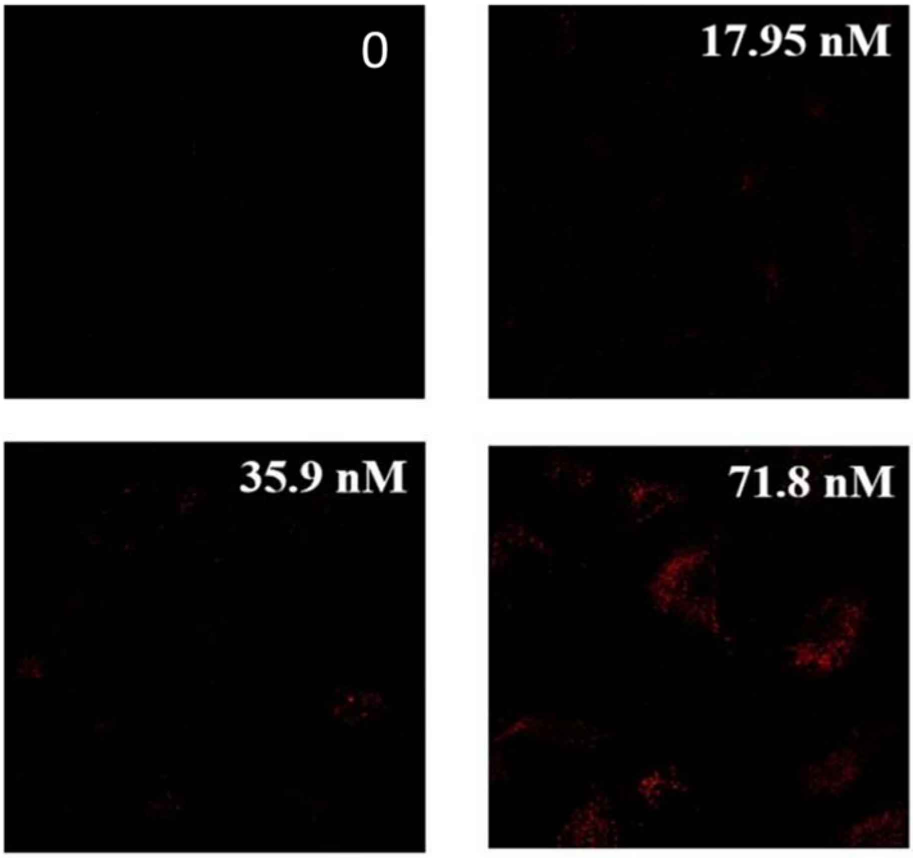

Figure S8. Confocal laser scanning microscopy image of Hela cells with endocytosed Au-PMA-TAMRA NPs after the exposure at different NP concentrations. The scale bar corresponds to 20 µm.

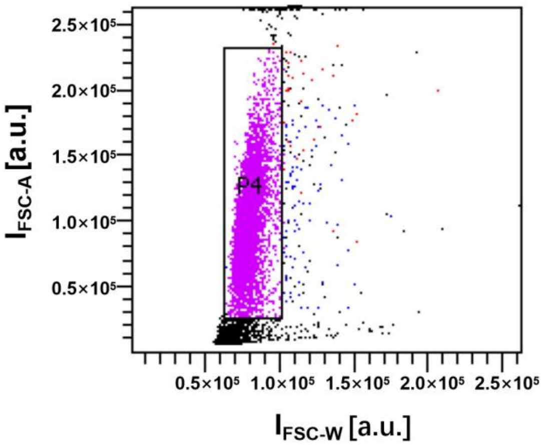

Figure S9. Density plot of the intensity of the forward-scattering area (I_{FSC-A}) versus the forward-scattering width (I_{FSC-W}) as recorded for Hela cells which had not been exposed to NPs and chloroquine. The box with the pink signal shows the gate ("P4") which was set to identify events of single Hela cells and this gate was used for all other measurements.

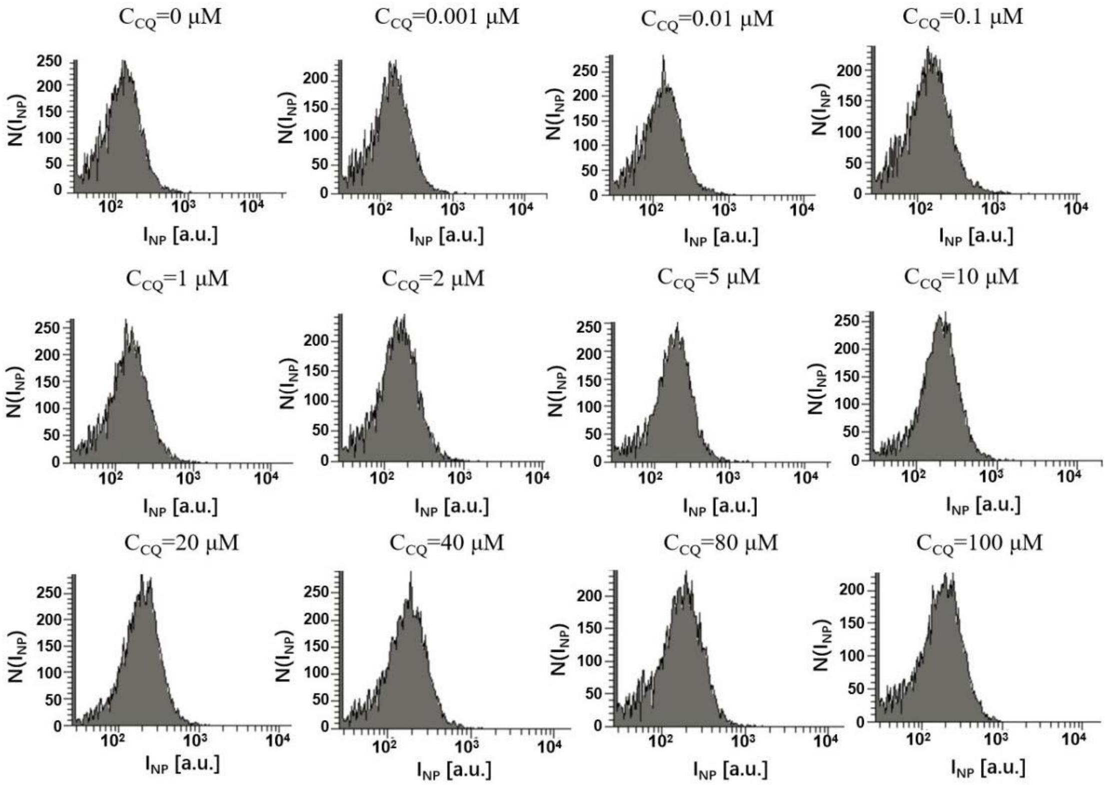

Figure S10. Flow cytometry analysis of Hela cells incubated for 24 h with different concentrations of chloroquine (without Au-PMA-TAMRA NPs). The fluorescence distribution in terms of the number $N(I_{NP})$ of counted NPs with a fluorescence intensity of I_{NP} of at least 10000 cells are shown.

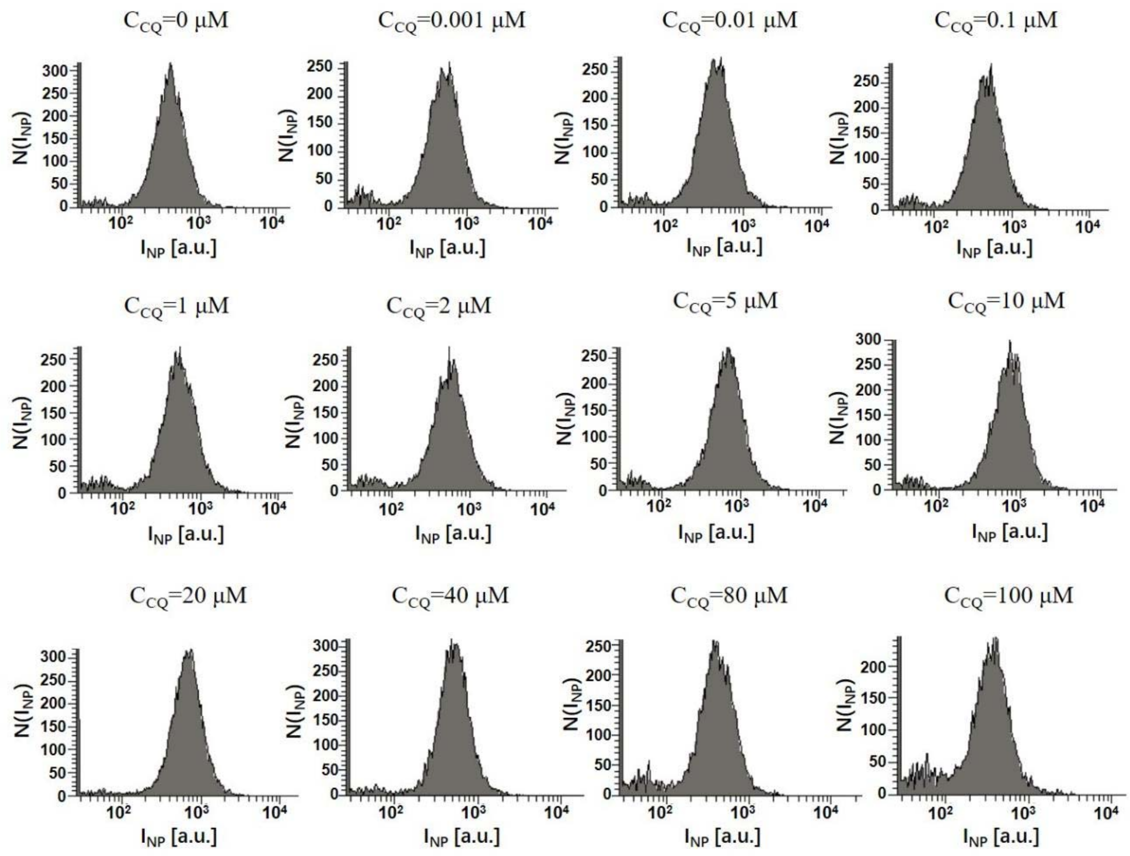

Figure S11. Flow cytometry analysis of Hela cells incubated for 24 h with different concentrations of chloroquine under the presence of Au-PMA-TAMRA NPs. The fluorescence distribution $N(I_{NP})$ of at least 10000 cells are shown.

6. Intracellular pH calibration curves for the pH-responsive capsules

The encapsulated SNARF has a ratiometric fluorescence emission, whereby the emission $I_{SNARF(y)}$ in the yellow increases for acidic pH, and the emission in the red $I_{SNARF(r)}$ increases for alkaline pH.[7, 8] Calibration curves for the ratio $I_{SNARF(r)}/I_{SNARF(y)}$ at different pH had to be recorded. As there may be scattering effects by cells, this calibration curve had to be recorded directly with capsules residing in endosomes/lysosomes, which were permeated in order to adjust the pH. This intracellular pH calibration curve was recorded as reported previously.[7] For this, HeLa cells were seeded in 24-well culture plates (Corning Costar, 1.9 cm^2 seeding area *per* well, 0.5 mL medium *per* well), with 3.0×10^4 cells *per* well, 24 h before experiments. Then, the cells were exposed to the pH-responsive capsules at a density of $N_{capsules/cell}$ = 7 capsules added *per* seeded cell. Cells were then incubated in culture medium for 24 h, after which they were washed twice using PBS followed by trypsination. After cell detachment trypsin was neutralized by adding complete culture medium (10% FBS), cells were pelleted by centrifugation, and the cell pellets were then resuspended in phosphate-citric acid buffer solutions of different pH. These solutions contained 20 μM of monensin and 10 μM of nigericin (for cell perforation), with a series of different pH values ranging from 3.0 to 8.0. After 15 min incubation the fluorescence intensities of these cells in the different pH buffers were measured using a FACScaliber flow cytometer (BD BioSciences) at with the excitation wavelength λ_{ex} = 561 nm. Red emission $I_{SNARF(r)}$ was collected with a bandpass filter of 670 ± 30 nm (PE-cy5 filter setting of the flow cytometer) and yellow emission $I_{SNARF(y)}$ was recorded with a bandpass filter =586 ± 15 nm (PE filter setting of the flow cytometer). The data were processed following a previous report,[7] using CellQuest software (BD BioSciences), see Figure S12 and Figure S13. Individual Hela cells were hereby identified with the "P5" gating. Note, that no control cell sample without added capsules was used, as due to shift of the capsule populations in the density plots fluorescence originating from capsules could be clearly separated from autofluorescence of cells. Then, the $I_{SNARF(r)}/I_{SNARF(y)}$ *versus* pH calibration curve was plotted, see Figure 2a. Note that the populations in the density plots of the flow cytometer data are likely not normal and the mean value might be skewed. For sake of simplicity we however limited our discussion to the mean values and did not consider the median values.

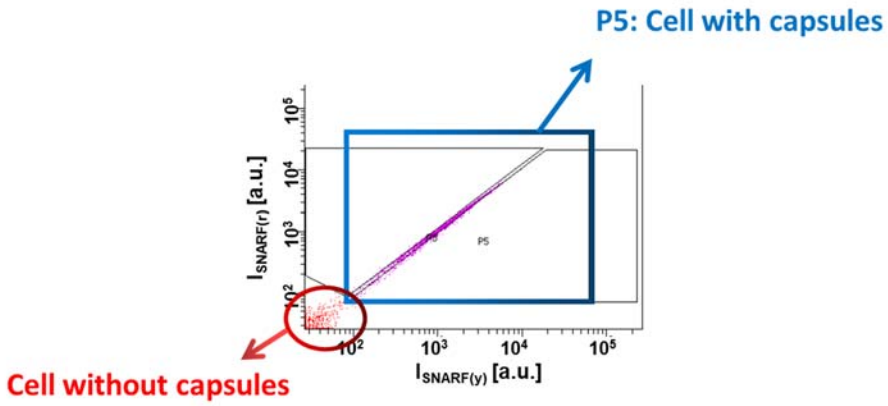

Figure S12. Gating strategy for recording the pH calibration curves shown in Figure 12. The "P5" gate was used to identify cells with associated capsules from cells without capsules. The "P5" gate was then applied for the data shown in Figure S13.

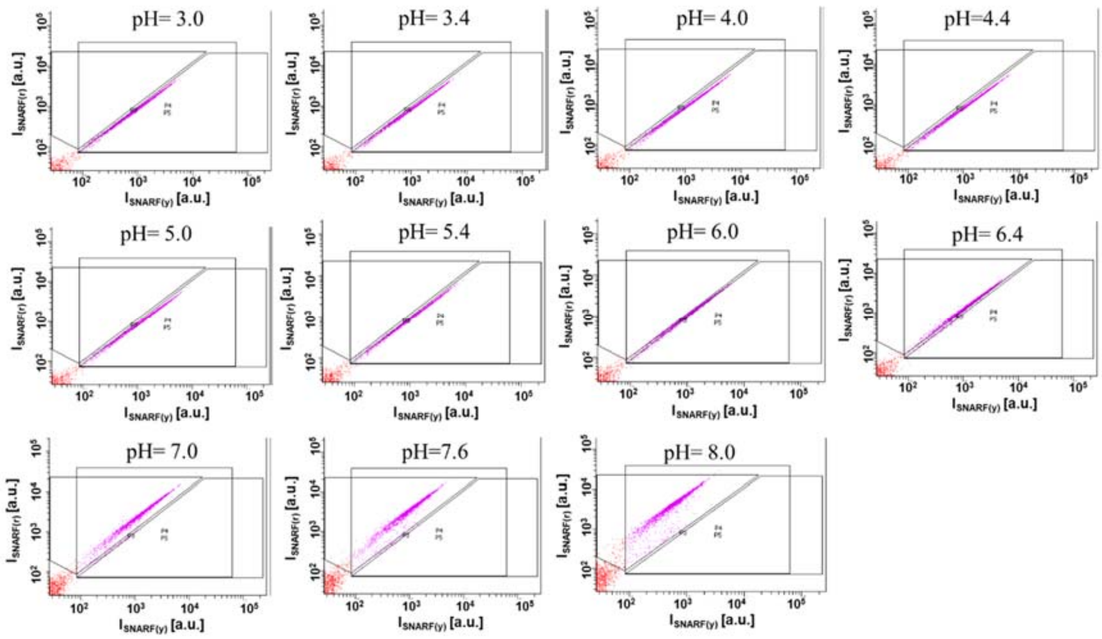

Figure S13. Density plots of the fluorescence intensities $I_{SNARF(r)}$ and $I_{SNARF(y)}$ of permeated cells with internalized pH-responsive capsules after incubation in a series of solutions with different pH values (3.0, 3.4, 4.0, 4.4, 5.0,5.4, 6.0, 6.4, 7.0, 7.6, and 8.0) containing 20 µM of monensin and 10 µM of nigericin.

7. Measurement of lysosomal pH at different chloroquine concentrations

With the calibration curve shown in Figure S13 and Figure 2a the pH in lysosomes of cells were measured, which were exposed to different chloroquine concentrations. HeLa cells were seeded in 24-well culture plates, with 3.0×10^4 cells *per* well, 24 h before experiments. Then, HeLa cells were exposed to pH-responsive capsules at a density of $N_{capsules/cell}$ = 7 added capsules *per* cell. Incubation was done in culture medium, containing different concentrations of chloroquine from 0 to 100 µM for 24 h. After this, cells were washes twice using PBS and then trypsinized. Thereafter, trypsin was neutralized by adding complete culture medium (10% FBS) and the detached HeLa cells pelleted by centrifugation and then resuspended in PBS. Subsequently, the fluorescence intensities of the resuspended cells were measured using a FACScaliber flow cytometer with the same settings as used for recording the calibration curve in Figure S13,[7] see Figure S14 and Figure S15. Using CellQuest software (BD BioSciences), the $I_{SNARF(r)}/I_{SNARF(y)}$ values of cells with capsules in lysosomes were calculated, and from these values the local lysosomal pH was the derived from the calibration curve shown in Figure 2a and are shown in Figure 2b₁, analogues to the description given for Figure S13.

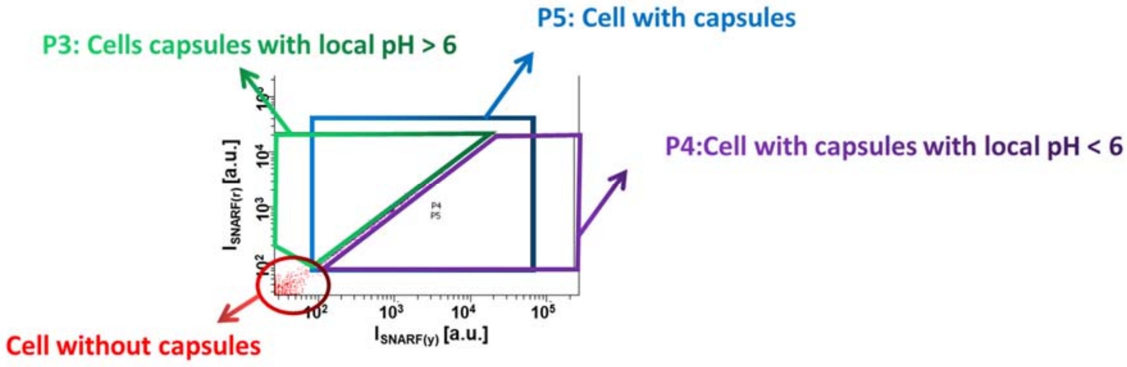

Figure S14. Gating strategy for recording the pH calibration curves shown in Figure 14. Based on the calibration curve shown in Figure S12 and S13, the "P4" gate was set to identify cells capsules whose pH of the local environment is < 6 (note, this is a different gate than then one shown in Figure S9). This is the case for capsules residing in endosomes/lysosomes. Cells with capsules with local pH > 6 were identified with gate "P3". The "P5" gate was then applied for the data shown in Figure S15. Note the chloroquine makes the pH in the lysosomes less acidic. At high doses of chloroquine, when the pH in the probed lysosome might be > 6, these events would not be considered, as they are outside "P4". Still, application of "P4" warrants for not considering events from capsules that are just adherent to the outer cell membrane and thus would not be inside lysosomes.

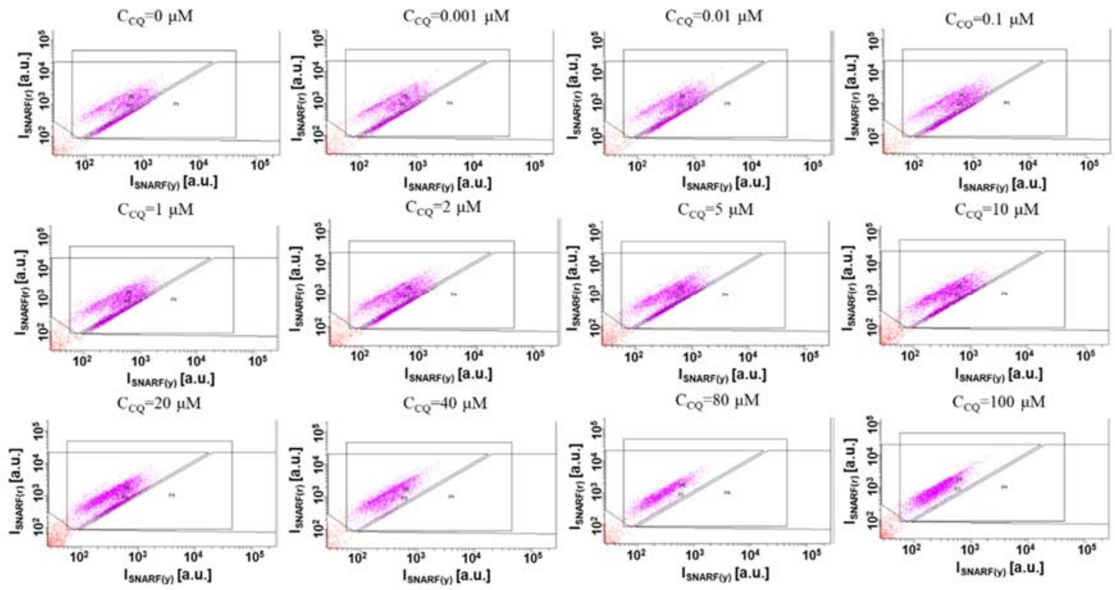

Figure S15. Density plots of the fluorescence intensities $I_{SNARF(r)}$ and $I_{SNARF(y)}$ of cells with internalized pH-responsive capsules after incubation in a series of solutions with different concentrations of chloroquine c_{CQ} from 0 to 100 µM for 24 h.

8. Gene transfection quantification

8.1. PEI/DNA complexes
8.2. Capsule/DNA complexes
8.3. Lipo2000/DNA complexes

8.1. PEI/DNA complexes

The transfection solution was prepared by dissolving PEI in 10 mM HEPES-NaOH buffer solution (pH = 7.4) to a concentration of 40 µg/mL, after which the PEI solution was added to an equal volume of peGFP solution (40 µg/mL). The resulting solution was vortexed for 5 s, and incubated for 20 min to form PEI/DNA complexes.[10]

HeLa cells were seeded in 24-well culture plates at 3.0×10^4 cells *per* well. After 24 h incubation at 37 °C and 5% CO_2, the cells were exposed to serum-free culture medium (500 µL) with different concentrations of chloroquine c_{CQ} (0, 0.001, 0.01, 0.1, 1, 2, 5, 10, 20, 40, 80, and 100 µM). One hour later, 12.5 µL transfection solution containing the PEI/DNA complexes was added into the culture medium. After 3 h, the media was then replaced with complete culture medium (10% FBS) again with different concentrations of chloroquine (0, 0.001, 0.01, 0.1, 1, 2, 5, 10, 20, 40, 80, and 100 µM). After 24 h incubation, the cells were washed twice using PBS, trypsinized, and after trypsin neutralization by adding complete culture medium (10% FBS) and centrifugation the pelleted HeLa cells were resuspended in PBS. The fluorescence intensities of the cells due to eGFP expression were measured using a FACScaliber flow cytometer (BD BioSciences, λ_{ex} = 488 nm, emission collected with a bandpass filter 530 ± 30 nm, corresponding to the FITC filter setting of the flow cytometer), see Figure S16. A minimum of 10000 cells was sampled for each condition. As control, the same series of measurements was performed, but without the addition of the PEI/DNA complexes, see Figure S17. The final results are plotted in Figure 3a_1.

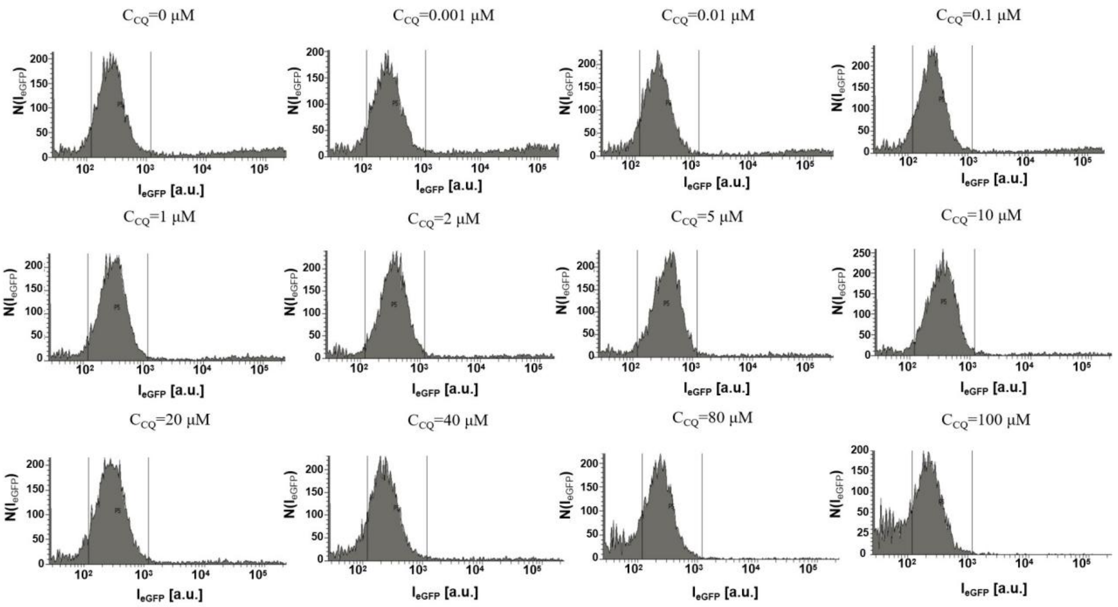

Figure S16. Flow cytometry analysis of Hela cells incubated with different concentrations of chloroquine together with added PEI/DNA complexes. The number of counted cells $N(I_{eGFP})$ with fluorescence intensity I_{eGFP} is shown.

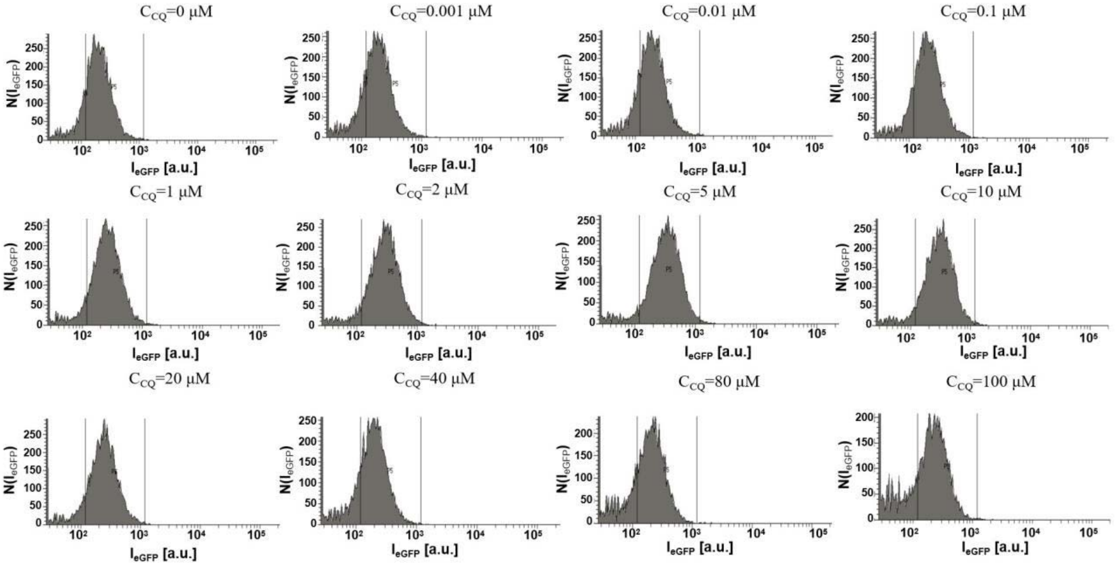

Figure S17. Flow cytometry analysis of Hela cells incubated with different concentrations of chloroquine (without the presence of PEI/DNA complexes). The number of counted cells $N(I_{eGFP})$ with fluorescence intensity I_{eGFP} is shown.

8.2. Capsule/DNA complexes

The transfection solution was prepared as reported previously.[11] Briefly, 33 μg of peGFP was diluted to a volume of 375 μL, and then 360 μL of $CaCl_2$ solution (0.5 M) was added. The obtained solution was added dropwise to 765 μL of Na_2CO_3 solution

(0.0063 M), the mixture was incubated for 5 min before transfection.

HeLa cells were seeded in 24-well culture plates with 3.0×10^4 cells *per* well. After 24 h incubation at 37 °C, 5% CO_2, the cells were exposed to complete culture medium (1000 µL) with different concentrations of chloroquine (0, 0.001, 0.01, 0.1, 1, 2, 5, 10, 20, 40, 80, and 100 µM). Then, after 30 min of incubation 25 µL transfection solution containing capsule/DNA complexes was added into the culture medium. After 24 h incubation the cells were washed twice using PBS, and then trypsinized to detach cells from the culture plates. Subsequently, trypsin was neutralized by adding complete culture medium (10% FBS), and cells were pellet by centrifugation. The cell pellet was resuspended in PBS. The fluorescence intensities of the cells were measured using flow cytometry are described for the PEI/DNA complexes, see Figure S18. As control a series with the same conditions, but without the addition of capsule/DNA complexes was done, see Figure S19. The final results are plotted in Figure 3b$_1$.

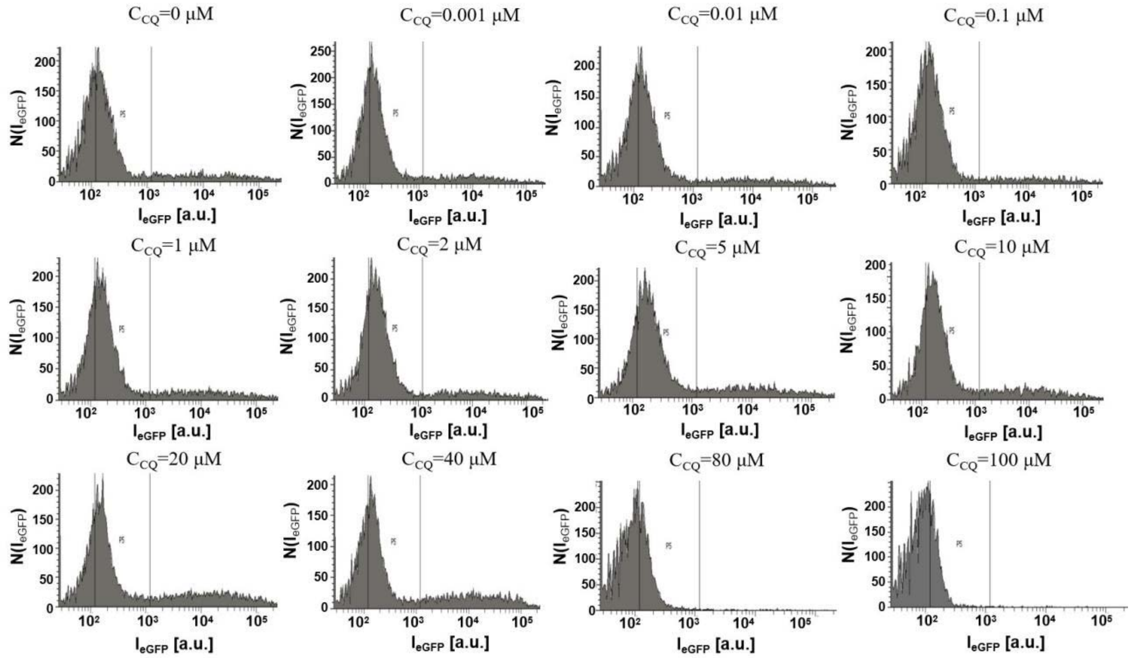

Figure S18. Flow cytometry analysis of Hela cells incubated with different concentrations of chloroquine under the presence of capsule/DNA complexes. The number of counted cells N(I_{eGFP}) with fluorescence intensity I_{eGFP} is shown.

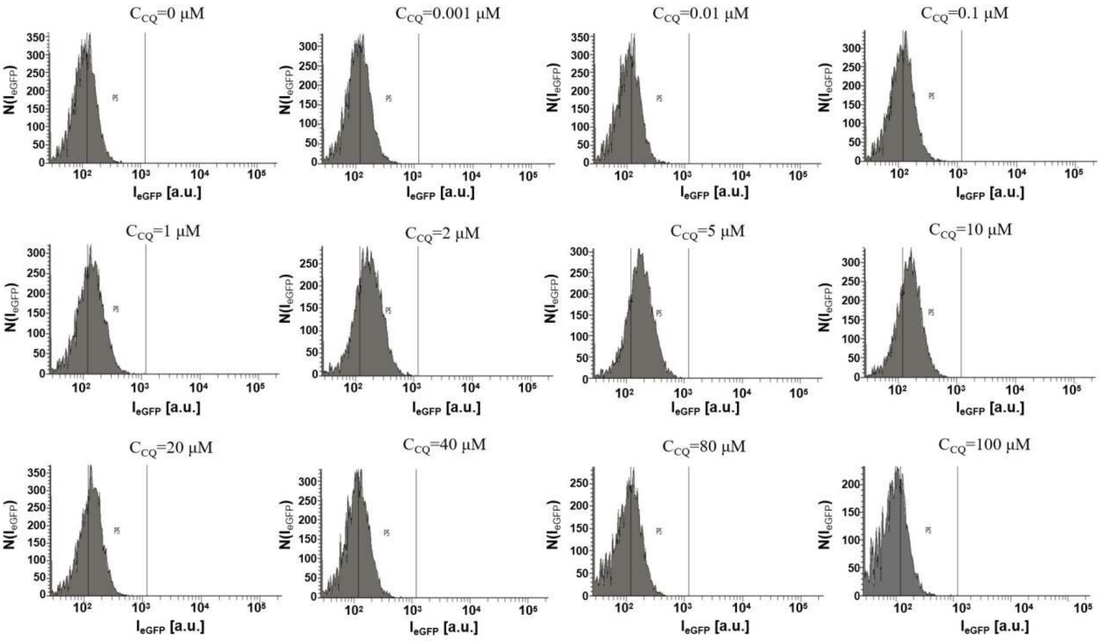

Figure S19. Flow cytometry analysis of Hela cells incubated with different concentrations of chloroquine (without the presence of capsule/DNA complexes). The number of counted cells $N(I_{eGFP})$ with fluorescence intensity I_{eGFP} is shown.

8.3. Lipo2000/DNA complexes

The transfection solution was prepared according to the manufacturer's protocol, see for example a related study.[12] In brief, 100 μL of serum-free DMEM containing 8 μL of Lipofectamine 2000 were mixed with 100 μL of serum-free DMEM containing 4.8 μg of peGFP, and the mixtures were incubated at room temperature for 5 min to obtain Lipo2000/DNA complexes. HeLa cells were seeded in 24-well culture plates with 3.0×10^4 cells *per* well. After 24 h incubation at 37 °C, 5% CO_2, the HeLa cells were exposed to complete culture medium (500 μL) with different concentrations of chloroquine (0, 0.001, 0.01, 0.1, 1, 2, 5, 10, 20, 40, 80, and 100 μM). Then, after 30 min of incubation 12.5 μL of transfection solution containing Lipo2000/DNA complexes was added. After 24 h incubation cells were washed twice using PBS, followed by trypsinized. Trypsin was neutralized by adding complete culture medium (10% FBS) and the detached cells were pelleted by centrifugation and then resuspended in PBS. The fluorescence intensities of the cells originating from expressed eGFP were measured using flow cytometry, see the description for the PEI/DNA complex analysis above, cf. Figure S20. As control a series with the same conditions, but without the addition of lipo2000/DNA complexes war done, see Figure S21. The final results are plotted in Figure 3c_1.

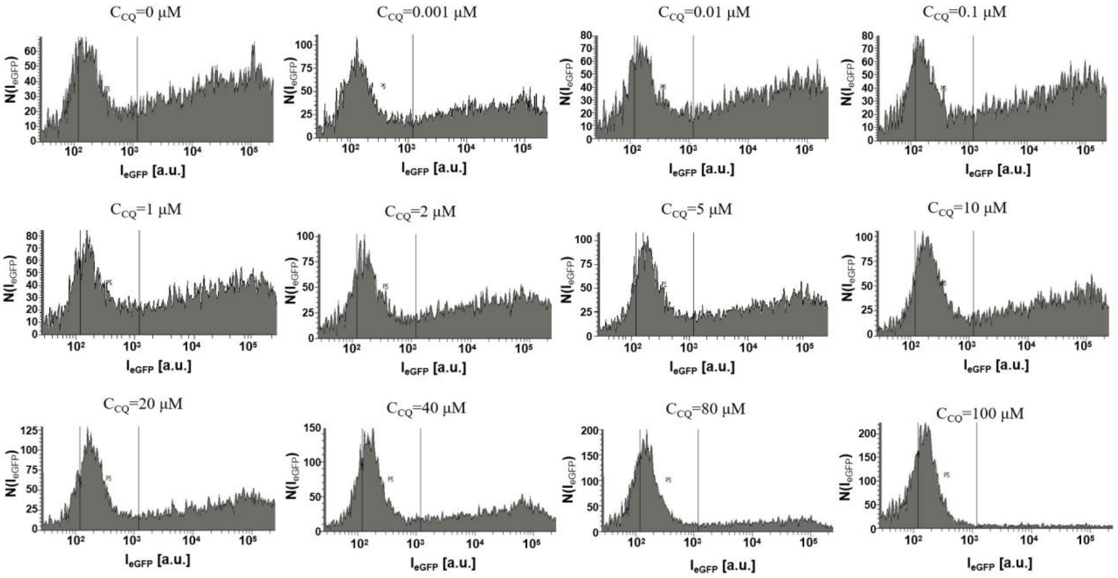

Figure S20. Flow cytometry analysis of Hela cells incubated with different concentrations of chloroquine under the presence of Lipo2000/DNA complexes. The number of counted cells $N(I_{eGFP})$ with fluorescence intensity I_{eGFP} is shown.

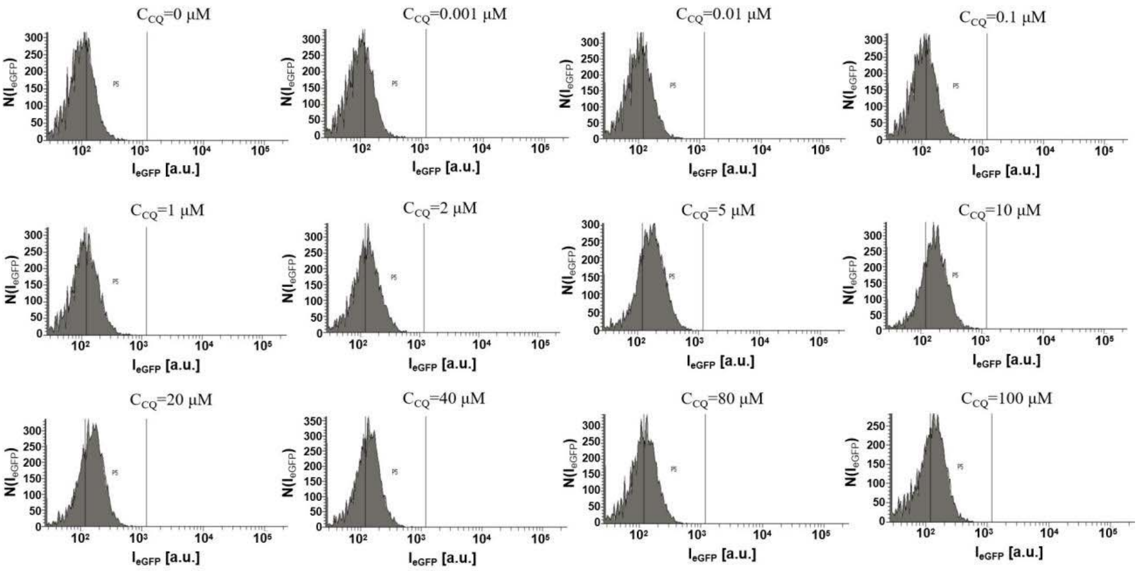

Figure S21. Flow cytometry analysis of Hela cells incubated with different concentrations of chloroquine (without the presence of Lipo2000/DNA complexes). The number of counted cells $N(I_{eGFP})$ with fluorescence intensity I_{eGFP} is shown.

9. References

1. Zhu, D. C.; Yan, H. J.; Zhou, Z. X.; Tang, J. B.; Liu, X. R.; Hartmann, R.; Parak, W. J.; Shen, Y. Q.; Feliu, N., Influence of the Modulation of the Protein Corona on Gene Expression Using Polyethylenimine (PEI) Polyplexes as Delivery Vehicle. *Advanced Healthcare Materials* **2021,** *20*, 2100125.
2. Hühn, J.; Carrillo-Carrion, C.; Soliman, M. G.; Pfeiffer, C.; Valdeperez, D.; Masood, A.; Chakraborty, I.; Zhu, L.; Gallego, M.; Zhao, Y.; Carril, M.; Feliu, N.; Escudero, A.; Alkilany, A. M.; Pelaz, B.; Pino, P. d.; Parak, W. J., Selected Standard Protocols for the Synthesis, Phase Transfer, and Characterization of Inorganic Colloidal Nanoparticles. *Chemistry of Materials* **2017,** *29*, 399−461.
3. Ma, X.; Hartmann, R.; Aberasturi, D. J. d.; Yang, F.; Soenen, S. J. H.; Manshian, B. B.; Franz, J.; Valdeperez, D.; Pelaz, B.; Feliu, N.; Hampp, N.; Riethmüller, C.; Vieker, H.; Frese, N.; Gölzhäuser, A.; Simonich, M.; Tanguay, R. L.; Liang, X.-J.; Parak, W. J., Colloidal Gold Nanoparticles Induce Changes in Cellular and Subcellular Morphology. *ACS Nano* **2017,** *11*, 7807−7820.
4. Cheng, J.; Zhang, Q.; Fan, S.; Zhang, A.; Liu, B.; Hong, Y.; Guo, J.; Cui, D.; Song, J., The vacuolization of macrophages induced by large amounts of inorganic nanoparticle uptake to enhance the immune response. *Nanoscale* **2019,** *11* (47), 22849-22859.
5. Riedinger, A.; Zhang, F.; Dommershausen, F.; Röcker, C.; Brandholt, S.; Nienhaus, G. U.; Koert, U.; Parak, W. J., Ratiometric Optical Sensing of Chloride Ions with Organic Fluorophore - Gold Nanoparticle Hybrids: A Systematic Study of Distance Dependency and the Influence of Surface Charge. *Small* **2010,** *6* (22), 2590-2597.
6. Rivera Gil, P.; Nazarenus, M.; Ashraf, S.; Parak, W. J., pH sensitive capsules as intracellular optical reporters for monitoring lysosomal pH changes upon stimulation. *Small* **2012,** *8* (6), 943-948.
7. Roy, S.; Zhu, D.; Parak, W. J.; Feliu, N., Lysosomal Proton Buffering of Poly(ethylenimine) Measured In Situ by Fluorescent pH-Sensor Microcapsules. *ACS Nano* **2020,** *14*, 8012-8023.
8. Hartmann, R.; Weidenbach, M.; Neubauer, M.; Fery, A.; Parak, W. J., Stiffness-dependent in vitro uptake and lysosomal acidification of colloidal particles. *Angewandte Chemie International Edition* **2015,** *54* (4), 1365-1368.
9. Chen, G.; Halim, H.; Yang, H.; Zhou, Y.; Zhu, D.; Parak, W. J.; Riedinger, A.; Feliu, N., Semiconductor Nanoplatelets as Ultra-Bright Fluorophores for Two-Photon Absorption Cell Imaging. *The Journal of Physical Chemistry* **2022,** *126*, 5658–5664.
10. Zhu, D.; Yan, H.; Zhou, Z.; Tang, J.; Liu, X.; Hartmann, R.; Parak, W. J.; Feliu, N.; Shen, Y., Detailed investigation on how the protein corona modulates the physicochemical properties and gene delivery of polyethylenimine (PEI) polyplexes. *Biomaterials Science* **2018,** *6*, 1800-1817.
11. Chen, S.; Zhao, D.; Li, F.; Zhuo, R.-X.; Cheng, S.-X., Co-delivery of genes and drugs with nanostructured calcium carbonate for cancer therapy. *RSC Advances* **2012,** *2* (5), 1820-1826.
12. Wong-Baeza, C.; Bustos, I.; Serna, M.; Tescucano, A.; Alcántara-Farfán, V.; Ibáñez, M.; Montañez, C.; Wong, C.; Baeza, I., Membrane fusion inducers, chloroquine and spermidine

increase lipoplex-mediated gene transfection. *Biochemical and Biophysical Research Communications* **2010,** *396* (2), 549-554.
